# Oceanographic regime and foraging behaviour structure compound-specific PFAS variability in arctic-atlantic guillemots

**DOI:** 10.1016/j.ese.2026.100707

**Published:** 2026-05-14

**Authors:** Rui Shen, Ralf Ebinghaus, Daniel Giddings Vassão, Norman Ratcliffe, Thomas Larsen

**Affiliations:** a Max Planck Institute of Geoanthropology, Jena, 07745, Germany; b Institute of Coastal Environmental Chemistry, Helmholtz Zentrum Hereon, Geesthacht, 21502, Germany; c Max Planck Institute for Chemical Ecology, Jena, 07745, Germany; d British Antarctic Survey, Natural Environment Research Council, Cambridge, CB3 0ET, UK; e Institute for Prehistoric and Protohistoric Archaeology, Christian-Albrechts-Universität zu Kiel, Kiel, 24118, Germany

**Keywords:** PFAS exposure variability, Water mass thresholds, Multi-tissue isotopes, Sympatric guillemots, Marine bioaccumulation

## Abstract

Current chemical exposures studies characterise chemical risk through mean-based concentrations, treating individual-level variability as statistical noise. However, this variability may carry structured ecological information that mean-based approaches systematically overlook. Here, we propose that individual per- and polyfluoroalkyl substance (PFAS) exposure variability constitutes a structured ecological signal, shaped by habitat use across oceanographic gradients and individual foraging behaviour, one that mean-based approaches are not designed to capture. To test the variability-as-signal hypothesis, we integrated two independent indices of individual stability using two sympatric guillemot species (*Uria aalge, n = 67* and *Uria lomvia, n = 45*) sampled across five Icelandic colonies during the 2018 breeding season. We paired PFAS variability scores, derived from plasma PFAS concentrations, with isotopic consistency scores derived from dual-tissue stable isotopes (δ^13^C and δ^15^N in plasma and red blood cells). These consistency scores represent individual foraging stability across the breeding season, enabling a reconstruction of foraging histories and oceanographic habitat use. Our results reveal that PFAS variability is highly structured by compound class, dominated by long-chain perfluoroalkyl carboxylic acids (PFCAs; 79% of variance) and perfluorooctane sulfonate (PFOS; 13%). Cluster analysis identified two main divergent exposure states: constrained PFOS variability versus constrained PFCA variability. Bivariate segmented regression revealed a hierarchical structure to contaminant acquisition: oceanographic regime (proxied by δ^13^C_consist_) functioned as the primary driver, with PFOS variability intensifying in Atlantic-influenced waters. Within these regimes, trophic sources (proxied by δ^15^N_consist_) emerged as a secondary, conditional modulator, specifically constraining PFCA variability among high-trophic individuals. At the colony scale, fine-scale niche partitioning, such as vertical foraging strategies and individual specialisation using glacial fjords and ice margins, produced compound-specific patterns that diverged from regional hierarchies. As climate change continues to redistribute Arctic and Atlantic water masses and reshape the food web structures, approaches that treat contaminant variability as ecological signal will be increasingly valuable for anticipating exposure regime shifts.

## Highlights

• PFAS variability constitutes a structured ecological signal of habitat use and foraging behaviours, not statistical noise.
• Isotopes reveal a hierarchy: water masses (δ^13^C) shape PFOS variability; trophic marker (δ^15^N) modulates PFCA variability.
• Atlantic foraging amplifies PFOS variability; elevated trophic positions constrain PFCA variability.
• Fine-scale niche partitioning likely generates localised variability patterns that diverge from regional hierarchies.

## Graphical abstract

**Figure:**
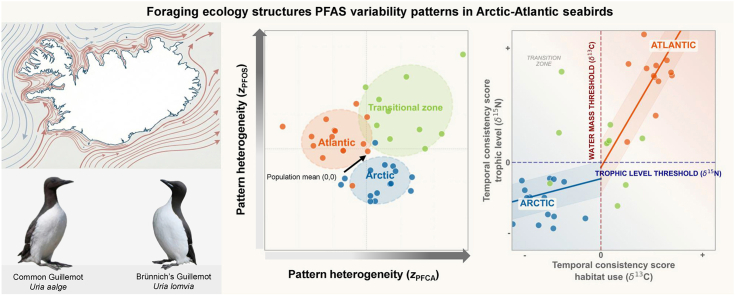

## 1 Introduction

Marine ecosystems, encompassing more than 90% of Earth's habitable biosphere volume [1], are threatened by multiple anthropogenic pressures [2], including the ubiquitous presence of synthetic chemical compounds such as per- and polyfluoroalkyl substances (PFAS) [3]. These persistent organic pollutants, characterised by stable carbon–fluorine bonds, are exceptionally resistant to environmental degradation [4] and readily accumulate in living organisms [5,6]. The toxic effects of PFAS pose measurable risks to both human and ecosystem health, particularly in marine environments where biomagnification can be pronounced through efficient trophic transfer from phytoplankton to apex predators [[6], [7], [8], [9]]. Conventional bioaccumulation studies primarily examine mean biomagnification trends, yet the variation around those means has received little analytical attention [10]. We propose that this variability, rather than representing stochastic noise, has an underlying ecological structure that records information about individual foraging consistency and habitat use, and that treating it as such opens a complementary approach to understanding PFAS dynamics.

The physicochemical properties of PFAS determine their bioaccumulation patterns in marine organisms [11,12]. Marine predator tissues are typically dominated by perfluorooctane sulfonate (PFOS) and long-chain perfluoroalkyl carboxylic acids (PFCAs) [3,7,13,14]. PFOS exhibits enhanced protein-binding affinity and prolonged retention due to its sulfonate group, which binds more strongly to serum proteins than the carboxylate groups of PFCAs [[15], [16], [17], [18]]. These molecular-level differences result in distinct exposure patterns in predators, with PFOS showing greater persistence and integrating exposure over longer time periods than PFCAs of comparable chain length [12,19]. This means that the two compound classes may respond differently to the same ecological gradient, and that the variability in tissue concentrations among individuals could, in principle, carry compound-specific ecological information. In practice, this variability has largely been treated as noise in bioaccumulation studies [6,8,20,21]. PFAS bioaccumulation research has traditionally relied on mean-based metrics such as trophic magnification factors and biomagnification factors [[20], [21], [22], [23], [24], [25]]. These are informative, but by averaging across individuals, time, and space they can obscure ecological variation [21]. Variability in PFAS tissue concentrations within and among populations can reflect underlying ecological processes - differences in prey choice, habitat use, and foraging consistency - that mean-based analyses may fail to capture. For example, low interindividual variability in seabirds may indicate specialised foraging within a consistent habitat, whereas high variability may reflect exposure to seasonally variable prey communities or spatially heterogeneous environments [26,27]. Recognising such patterns becomes increasingly important as climate change reshapes marine ecosystems and alters predator foraging behaviour and contaminant exposure dynamics.

Spatial patterns of PFAS distribution in marine ecosystems reflect interactions between ecological and oceanographic processes [28]. Previous research has primarily attributed these patterns to long-range transport and geographic variability [[29], [30], [31], [32], [33]]. However, distribution gradients also arise from differences in food web structure and habitat dynamics [34]. In Arctic waters, PFAS exposure tends to be relatively homogeneous within species because individuals are constrained to narrow, well-segregated habitats and rely on similar, energy-rich prey, thereby reducing interindividual variation in contaminant uptake. These systems typically exhibit more modular, less connected food webs than those in temperate regions [26,35]. In contrast, Atlantic waters support more diverse prey assemblages, including a range of forage fish and mesopelagic fauna, with greater connectivity and broader feeding opportunities [36]. These differences in food web complexity and connectivity are expected to produce greater interindividual variation in contaminant exposure in Atlantic-influenced birds than in Arctic-influenced ones.

Seabirds, as central-place foragers during the breeding season, provide an ideal model for exploring these dynamics [37,38]. This period offers an ideal ecological context for examining PFAS exposure variability because central-place foraging constrains individuals to localised ranges for chick provisioning [[39], [40], [41], [42]]. As a result, central-place foraging can promote more homogeneous exposure patterns among individuals, independent of whether breeding occurs in Arctic or temperate environments [43,44]. Two sympatric seabird species—Brünnich's guillemots (*Uria lomvia*, UL) and common guillemots (*Uria aalge*, UA) illustrate this dynamic. Despite overlapping ecological niches, UL generally prefer more lipid-rich prey and colder foraging habitats than UA [45]. Outside the breeding season, both species disperse across diverse habitats and prey communities [46,47], potentially increasing heterogeneity in contaminant exposure. By focusing on the breeding season of these two species, this study uses the ecological consistency imposed by central-place foraging to disentangle how spatial habitat use and trophic interactions shape PFAS exposure patterns.

Iceland's coastal waters, located at the confluence of cold Arctic and warm Atlantic currents, provide a unique natural laboratory for investigating PFAS exposure in marine ecosystems [[48], [49], [50]]. This region lies at the boundary between Arctic and temperate waters, creating distinct oceanographic and ecological zones [51]. To the north, cold Arctic waters with seasonal sea ice coverage generally exhibit lower primary productivity than temperate waters, except for localised hotspots such as ice edges and polynyas [51,52]. These Arctic systems experience pronounced seasonal production, typically limited to a one-to four-month period during spring and summer [53], resulting in highly seasonal trophic dynamics [54]. In contrast, the warmer Atlantic-influenced waters to the south lack sea ice and generally support higher and more consistent primary productivity [51,55]. Around Iceland, Atlantic-influenced ecosystems tend to have more complex, stable, and highly connected food webs than Arctic systems because the mixing of Arctic and Atlantic water masses introduces a broader range of prey species and ecological niches [36,56]. This convergence creates sharp ecological transitions, with abrupt shifts in isotopic baselines (δ^13^C, δ^15^N) and prey communities across water masses, providing an opportunity to test how oceanographic regimes structure PFAS variability patterns.

To assess individual-level foraging consistency, we used stable isotope analysis of two blood fractions with different turnover rates. Plasma integrates dietary information over short periods (approximately 1 week), whereas red blood cells reflect longer timeframes (approximately 3–4 weeks) [57]. By combining isotopic values from these tissues, we evaluated how temporal consistency in foraging behaviour shapes individual PFAS exposure patterns. This dual-tissue approach provides a more detailed understanding of how individual foraging strategies translate into PFAS variability patterns than single-tissue analysis can.

We reconceptualise PFAS exposure variability as an ecological signal rather than statistical noise to explore contaminant dynamics in marine ecosystems. Using Iceland's position at the Arctic–Atlantic interface, we hypothesize that PFAS exposure variability reflects structured ecological signals driven by species-specific foraging strategies and oceanographic conditions. Specifically, we predict that UL, which preferentially forage in Arctic waters and exhibit more specialised feeding behaviour, will yield a more constrained and lower PFAS variability, manifesting as low intra-population variance; in contrast, UA, with broader habitat use and more generalist foraging behaviour, will result in greater inter-individual exposure heterogeneity.

## 2 Methods and materials

### 2.1 Study design

**Fig. 1:**
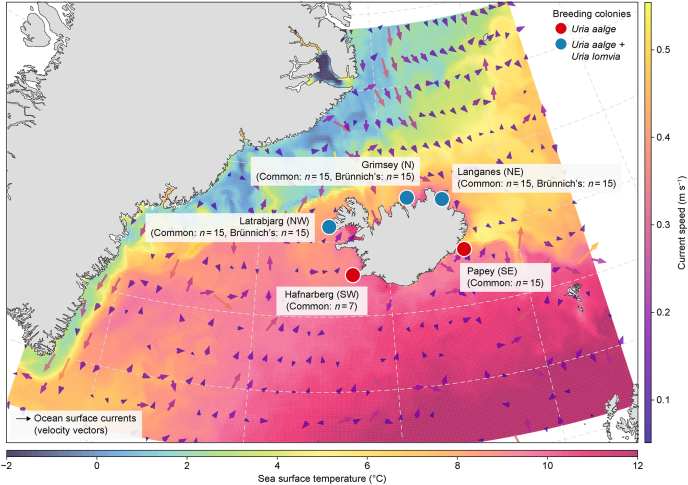
**Sea-surface temperature and guillemot sampling sites around Iceland (June 2018).** Sea surface temperature (SST) is shown alongside the major ocean currents influencing Icelandic waters: the warm Irminger and North Atlantic Currents, and the cold East Greenland and East Icelandic Currents. Guillemots were sampled at five colonies. Látrabjarg (northwest [NW]; 65.50° N, 24.52° W), located at the interface between the warm Irminger Current and the cold East Greenland Current; Grímsey Island (north [N]; 66.57° N, 18.02° W), primarily influenced by the warmer waters of the Irminger Current with some contribution from the cooler East Icelandic Current; Langanes peninsula (northeast [NE]; 66.38° N, 14.54° W), where the Irminger and East Icelandic Currents meet; Papey (southeast [SE]; 64.59° N, 14.18° W), situated in the cool East Icelandic Current, near its convergence with the warm North Atlantic Current; and Hafnaberg (southwest [60]; 63.75° N, 22.75° W), affected by the warmer waters of the North Atlantic Current. The three northern colonies (NW, N, and NE) host both common guillemots (*Uria aalge*, UA) and Brünnich's guillemots (*Uria lomvia*, UL). The NW colony is the largest colony (343,900 guillemot pairs; UA:UL = 1.9:1.0), and the N colony is the second largest (71,400 pairs; UA:UL = 16.4:1.0) as per a 2008 census [61]. Only UA breeds in the two southern colonies (SE and SW). SST data are from Copernicus Marine Environment Monitoring Service Global Ocean Physics Reanalysis [62].

We analysed 112 blood samples from UA (*n* = 67) and UL (*n* = 45) collected at five colonies across Iceland during June and July 2018 (Fig. 1) [58]. Sampling locations encompassed diverse oceanographic influences along both north–south and east–west gradients. Published stable isotope data from the region indicate distinct spatial patterns: δ^13^C values follow a latitudinal gradient, with higher values in Atlantic-influenced waters than in Arctic waters, while δ^15^N values reflect both trophic position and local oceanographic conditions [59].

The three northernmost colonies (NW, N, NE) harboured both UA and UL populations, whereas the two southernmost colonies (SE, SW) hosted UA only. At the three northernmost colonies where both species coexist, we collected 15 samples per species per colony (*n* = 45 for UL and *n* = 45 for UA in total). At the two southernmost colonies, where only UA breeds, we collected 15 samples at SE and seven samples at SW due to logistical constraints. Blood samples were separated into cells and plasma to evaluate guillemot foraging across different temporal scales. PFAS concentrations were measured in plasma because PFAS bind strongly to albumin, a major plasma protein.

The presence of both species at the northernmost colonies enabled direct interspecies comparisons of PFAS exposure under shared oceanographic conditions. The balanced sampling design at the northernmost colonies (*n* = 15 per species) provided sufficient power for such comparisons. Migration patterns differ between species: UL undertake extensive post-breeding migrations from high-latitude breeding sites to lower-latitude wintering areas, with birds from the NW colony typically wintering in Southwest Greenland and those from the N and NE colonies wintering off West Greenland and north of Iceland [63]. In contrast, UA generally winter closer to their breeding colonies, although some individuals migrate to the Mid-Atlantic Ridge or waters between the Faroe Islands, Scotland, and Shetland Islands [64].

### 2.2 Sample collection

To ensure a representative cross-section of Icelandic seabird populations, blood samples were collected from selected breeding colonies as described in Bonnet-Lebrun [65]. Approximately 1 mL of blood was collected from each seabird via venipuncture, using rapid, minimally invasive sampling techniques to ensure animal welfare. Following collection, blood samples were centrifuged using a Micro Star 12 centrifuge (VWR, Leuven, Belgium) at 12,300 rpm for 4 min to separate plasma and cells. The separated fractions were transferred to glass petri dishes and dried at ambient temperature in a desiccator for four to six days. Sample preparation followed standardized protocols (Supplementary Method Section 1.1).

### 2.3 PFAS chemical analysis

PFAS analysis was conducted on plasma samples at the Helmholtz-Zentrum Hereon (Geesthacht, Germany). The targeted analysis included 14 PFAS: nine PFCAs (C5–C13), four perfluoroalkyl sulfonic acids (PFSAs: C4, C6, C8, C10), and hexafluoropropylene oxide dimer acid (HFPO-DA) (Supplementary Table S1). PFAS were extracted from dried plasma samples using a modified quaternary ammonium salt-based ion-pairing method [66,67]. Prior to extraction, samples were fortified with ^13^C-labelled PFAS internal standards and buffered with sodium carbonate (1 mL, 0.5 M, pH 10.0). An analytical procedure was performed according to a detailed extraction protocol involving specific solvent grades and chemical reagents (Supplementary Table S2; Supplementary Method Section 1.3).

Analysis was performed using high-performance liquid chromatography–tandem mass spectrometry (HPLC–MS/MS) with electrospray ionization in multiple-reaction monitoring mode (Supplementary Table S3–S4). Chromatographic separation was achieved using a Synergi Fusion-RP C18 column with a water–methanol gradient containing ammonium acetate buffer. Method detection limits ranged from 0.05 to 0.07 ng mL^−1^ for all target compounds (Supplementary Table S6). PFAS concentrations are reported on a dry-weight basis (ng g^−1^ DM), consistent with the dried sample preparation approach. Detailed analytical parameters, quality assurance, and quality control procedures are documented in the supplement (Supplementary Table S5–S6; Supplementary Method Section 1.5).

### 2.4 Stable isotope analysis

Stable isotope data for blood cells reported here were previously published by Bonnet-Lebrun [65], whereas plasma isotope data are presented for the first time in this study. Isotope values are expressed in delta (δ) notation as follows:

$$\delta =\left(\frac{R_{\text{sample}}}{R_{\text{standard}}}-1\right)\times 1000$$

where *R*_sample_ is the ratio of the heavy to light isotope in the sample, and *R*_standard_ is the corresponding ratio in the international standard. Isotopic values are expressed in per mil (‰) relative to Vienna Pee Dee Belemnite for carbon and atmospheric air for nitrogen.

Elemental content and bulk isotope values of blood cells were prepared following Bonnet-Lebrun [65] and analysed at the Stable Isotope Facility of the Experimental Ecology Group, GEOMAR (Kiel, Germany), using a customized high-sensitivity elemental analyser coupled to a stable isotope ratio mass spectrometer (DeltaPlus Advantage, Thermo Fisher Scientific, Germany), as described by Hansen and Sommer [68]. Analytical precision (standard deviation) was 0.15‰ for δ^13^C and 0.25‰ for δ^15^N (*n* = 3).

### 2.5 Statistical analysis

All statistical analyses were conducted in Python 3.9.16 using Pandas 1.5.3, NumPy 1.24.3, SciPy 1.10.1, Scikit-learn 1.2.2, Statsmodels 0.13.5, Matplotlib 3.7.1, and Seaborn 0.12.2. The analytical workflow consisted of four sequential steps designed to characterise PFAS variability patterns and their relationships with ecological indicators.

First, PFAS concentration data were log-transformed to improve normality. Principal component analysis (PCA) was then performed to reduce dimensionality and identify major patterns of covariation among PFAS across individual birds. Based on PCA results (Supplementary Fig. S1), individual PC scores were standardized into *z*-scores to quantify the degree to which each bird expressed these covariation patterns. A *z*-score of zero represents the population mean. Negative *z*-scores indicate more constrained (consistent) exposure patterns, whereas positive *z*-scores indicate more variable (heterogeneous) exposure patterns. Importantly, the interpretation of these *z*-scores is specific to the PCA loading structure observed in this dataset and is used here to distinguish individuals with constrained versus variable PFAS exposure profiles that may reflect different foraging strategies.

Second, *K*-means clustering was used to characterise population-level patterns in PFAS variability. The optimal number of clusters was determined using silhouette analysis, which maximizes within-cluster similarity and between-cluster separation. Silhouette scores range from −1 to +1, with higher values indicating better cluster assignment.

Finally, to assess potential threshold effects in relationships between isotopic consistency and PFAS variability patterns, bivariate segmented regression was applied. Isotopic consistency scores (δ^13^C_consist_ and δ^15^N_consist_) were calculated using PCA to capture shared consistency between tissues, followed by standardization into *z*-scores. A consistency score of zero represents the population mean, with positive and negative values indicating consistently higher or lower isotopic values across tissues, respectively. The absolute magnitude of these scores reflects how strongly an individual's isotopic pattern deviates from the population mean across both temporal scales.

To quantify oceanographic influences on PFAS variability, we developed a Bivariate Segmented Regression model (Supplementary Section 2.4). We utilised isotopic functional breakpoints (*ψ*) to characterise the transition between distinct ecological regimes, using foraging consistency metrics (δ^13^C_consist_ and δ^15^N_consist_) (Supplementary Section 2.3) to map the isotopic landscape. Uncertainty was estimated for each foraging quadrant individually, rather than averaged globally. Standard deviations for each regime were derived from Median Absolute Deviation-rescaled residuals (Supplementary Section 2.4), generating distance-weighted 95% confidence intervals. The exposure surface was parameterised using the identified breakpoints and segment-specific slopes, predicting PFAS variability across the bivariate isotopic landscape (Supplementary Section 2.5). To minimise the risk of overfitting and ensure the generalisability of our findings, we implemented a twofold validation strategy. First, isotopic thresholds were fixed rather than being optimised to the specific dataset. Second, the dataset was partitioned into training (80%) and testing (20%) subsets. Model performance was evaluated on the held-out testing data using the coefficient of determination (*R*^2^) and root-mean-square error (*RMSE*). Statistical inference for the estimated coefficients, including the segment-interaction terms, was derived from the variance-covariance matrix to calculate robust standard errors and two-tailed *p*-values (*α* = 0.05) (Supplementary Section 2.6).

## 3 Results

### 3.1 PFAS variability patterns

**Fig. 2:**
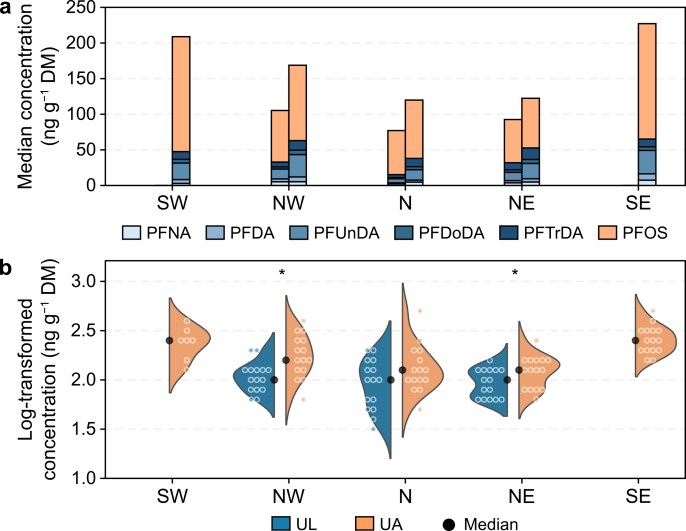
**PFAS composition and concentrations in Icelandic guillemots across species and colonies.** Per- and polyfluoroalkyl substance (PFAS) profiles are compared between Brünnich's guillemots (*Uria lomvia,* UL) and common guillemots (*Uria aalge*, UA) across five colonies in Iceland (NW [northwest], N [north], NE [northeast], SW [southwest], and SE [southeast]). **a**, Median PFAS concentrations of five long-chain perfluoroalkyl carboxylic acids (C9–C13 PFCAs) and perfluorooctane sulfonate (PFOS, linear and branched). Left: UL; right: UA. **b**, Log-transformed PFAS concentrations (including C9–C13 PFCAs and PFOS) shown as split-violin plots. Each data point represents an individual bird, while the split-violin shapes reflect the overall distribution for each colony and species. Black dots indicate median values for each species at each colony, and asterisks represent significance levels between species at the sympatric colony (∗*p ≤* 0.05).

Analysis of PFAS concentrations revealed consistent exposure patterns across both guillemot species (UA and UL). Long-chain PFCAs (C9–C13) and PFOS (linear and branched) were the dominant compounds detected in all samples. PFOS accounted for 62–73% of the total PFAS burden in UA and 70–89% in UL. Among PFCAs, PFUnDA (C11) and PFTrDA (C13) were the most abundant, with median concentrations ranging from 10.0 to 23.3 ng g^−1^ DM in UA and 6.6 to 10.0 ng g^−1^ DM in UL. Short-chain PFCAs (e.g., PFPeA, PFHxA), PFBS, and HFPO-DA were not detected in any samples. Detection frequencies and concentration ranges were identified across all PFAS, with median PFAS concentrations illustrated by species and colony (Table 1; Fig. 2).

**Table 1:** Detection rates and concentration ranges of per- and polyfluoroalkyl substances (PFAS) in Brünnich's guillemots (*Uria lomvia*, UL) and common guillemots (*Uria aalge*, UA).

Compound	UL				UA			
	DF	Median	Min.	Max.	DF	Median	Min.	Max.
	(%)	(ng g^−1^ DM)			(%)	(ng g^−1^ DM)		
PFPeA	n.d.	<MDL	<MDL	<MDL	n.d.	<MDL	<MDL	<MDL
PFHxA	n.d.	<MDL	<MDL	<MDL	n.d.	<MDL	<MDL	<MDL
PFHpA	49	0.1	<MDL	0.9	27	0.1	<MDL	0.4
PFOA	47	0.4	<MDL	1.8	40	0.5	<MDL	2.8
PFNA	100	3.4	0.9	16.5	100	4.4	1.1	24.2
PFDA	100	2.7	0.4	15.5	100	5.6	1.0	21.5
PFUnDA	100	10.0	1.3	48.1	100	23.3	5.9	95.5
PFDoDA	100	2.4	0.3	9.3	100	5.0	1.6	17.9
PFTrDA	100	6.6	1.2	20.4	100	12.9	4.2	38.7
PFBS	33	<MDL	<MDL	0.1	21	<MDL	<MDL	0.2
PFHxS	27	0.4	<MDL	1.2	40	0.4	<MDL	1.7
PFHpS	7	1.9	0.6	1.9	4	2.2	1.6	5.0
PFOS	100	69.8	22.8	203	100	103	23.1	409.7
PFDS	69	1.5	<MDL	12.7	70	0.7	<MDL	7.0
HFPO-DA	n.d.	<MDL	<MDL	< MDL	n.d.	<MDL	<MDL	<MDL

DF, detection frequency; DM, dry mass; MDL, method detection limit; n.d.: not detectable.

PCA identified two primary axes of PFAS variability. The first principal component (PC1), driven mainly by long-chain PFCAs (C9–C13), explained 79% of the total variance. The second component (PC2), dominated by PFOS and varying independently from the other compounds, accounted for an additional 13% of the variance (Supplementary Fig. S1). Based on these patterns, *z*-scores were calculated for PFCA-driven variability (*z*_PFCA_) and PFOS-driven variability (*z*_PFOS_) to quantify individual-level expression of each exposure pattern.

*z*_PFCA_ values ranged from −2.3 to 2.9 across all individuals. Northernmost UA exhibited predominantly negative *z*-scores (median: −0.6 to 0.2), indicating tighter clustering around the regional mean and reduced inter-individual variability. In contrast, the northernmost UL showed predominantly positive values (median: 0.3–1.1), reflecting greater variability. *z*_PFOS_ values ranged from −2.1 to 3.4. Southernmost UA populations exhibited elevated positive *z*-scores (median: 0.7–0.8), indicating high variability in PFOS exposure (maximum values, SW: 3.4; SE: 2.7). Northernmost populations of both species showed more constrained PFOS patterns, with predominantly negative *z*-scores across colonies (median, UA: −0.7 to −0.2; UL: −0.5 to 0.4).

**Fig. 3:**
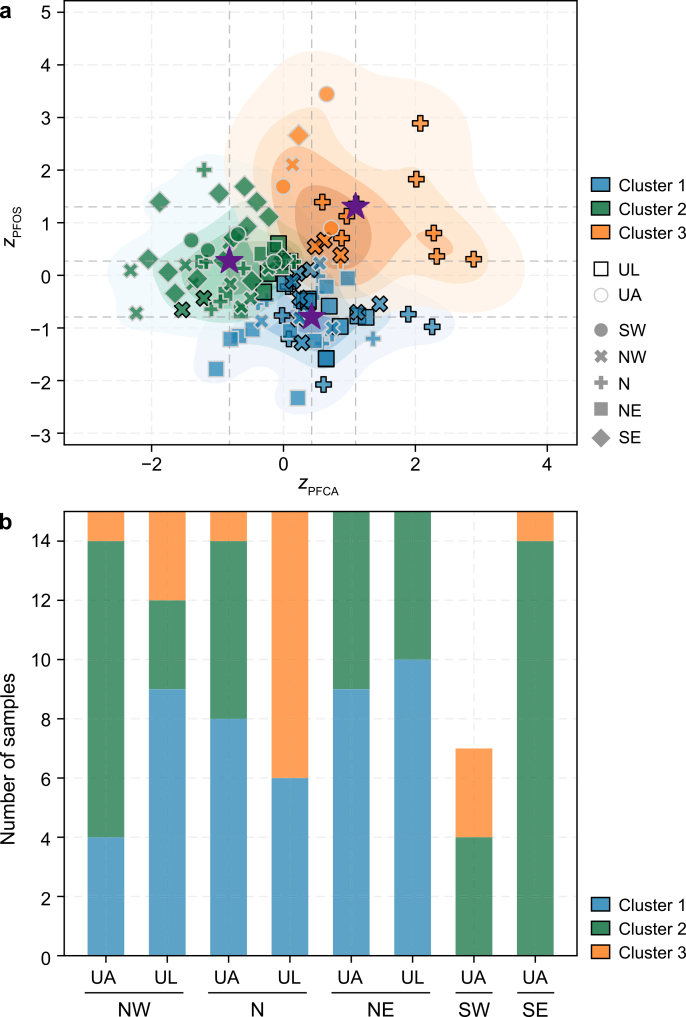
**Clustering of PFAS variability in common and Brünnich's guillemots. a**, *K*-means clustering based on standardised variability (*z*-scores) in long-chain PFCAs (*z*_PFCA_, C9–C13) and PFOS (*z*_PFOS_). Shaded underlying regions represent Kernel Density Estimation contours for each cluster, illustrating the probability density of observations; darker internal shades indicate areas of highest data density. The zero point on each axis represents the mean variability, with positive and negative values indicating higher and lower variability than the mean, respectively. Each point represents an individual seabird, with edge colours denoting species (black: *Uria lomvia* [UL]; white: *Uria aalge* [UA]), shapes indicating colony location, and fill colours representing cluster assignment. Purple stars mark cluster centroids. b, Colony-level distribution of cluster membership, shown as the proportion of individuals from each species assigned to each cluster at each colony. Colony codes: NW, northwest; N, north; NE, northeast; SW, southwest; and SE, southeast.

To categorise birds based on their PFAS exposure variability profiles, we performed *k*-means clustering. The *k*-means model (*k* = 3) identified three distinct PFAS variability patterns across guillemot populations (Fig. 3), with an overall silhouette score of 0.4. The first cluster (*n* = 46; silhouette score: 0.4) was centred at *z*_PFCA_ = 0.4 ± 0.6 and *z*_PFOS_ = −0.8 ± 0.6, indicating divergent PFCA variability combined with constrained PFOS variability. The second and largest cluster (*n* = 48; silhouette score: 0.3) exhibited the opposite pattern, with consistently low PFCA variability but high PFOS variability (*z*_PFCA_ = −0.8 ± 0.6; *z*_PFOS_ = 0.3 ± 0.6). The smallest cluster (*n* = 18; silhouette score: 0.3) showed elevated variability in both PFAS classes, with strongly positive values for both metrics (*z*_PFCA_ = 1.1 ± 0.8; *z*_PFOS_ = 1.3 ± 0.9).

PFAS variability patterns showed a clear north–south gradient. Cluster 1 (divergent PFCA variability, low PFOS variability) dominated northern colonies, while Cluster 2 (low PFCA variability, divergent PFOS variability) was most prevalent in southern colonies. Species composition varied by location. At the NW colony, UA individuals were primarily assigned to Cluster 2, whereas UL individuals were mainly assigned to Cluster 1. At the N colony, both species were largely represented in Cluster 1 but diverged in secondary cluster membership, with UA grouping mainly in Cluster 2 and UL in Cluster 3. At the NE colony, both species overwhelmingly occupied Cluster 1, showing minimal interspecies divergence.

### 3.2 Isotopic consistency across tissues

**Fig. 4:**
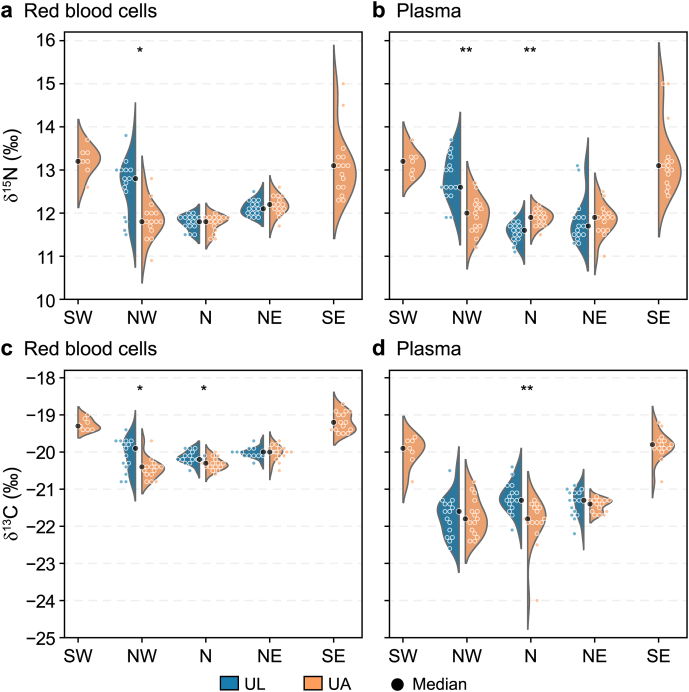
**Stable isotope composition of guillemot tissues across Icelandic colonies.** Split violin plots show the distribution of carbon (δ^13^C; **a**, **b**) and nitrogen (δ^15^N; **c**, **d**) isotope values in red blood cells (**a**, **c**) and plasma (b, d) from Brünnich's guillemots (*Uria lomvia*, UL) and common guillemots (*Uria aalge*, UA) across five geographic colonies: southwest (SW), northwest (NW), north (N), northeast (NE), and southeast (SE). Violin plots display kernel density estimates with individual data points overlaid; black dots represent the median. Asterisks denote statistical significance of between-species comparisons at sympatric colonies (∗*p* < 0.05, ∗∗*p* < 0.01).

Red blood cell δ^13^C values exhibited a pronounced north–south gradient, ranging from −22‰ to −19‰, with more negative values in northern colonies and more positive values in southern colonies (Fig. 4). Plasma δ^13^C values followed a similar spatial pattern but were slightly more negative overall (Fig. 4). Red blood cell δ^15^N values ranged from 11‰ to 14‰, while plasma δ^15^N values ranged from 11‰ to 15‰. Both tissues showed the highest δ^15^N values at the SE colony. Stable isotopic values varied by group, with specific medians and interquartile ranges detailed in Supplementary Table S7.

To assess foraging consistency during the breeding season, we examined isotopic relationships between plasma and red blood cells. Significant positive correlations were observed for both δ^13^C (*r* = 0.7, *p* < 0.001) and δ^15^N (*r* = 0.7, *p* < 0.001), indicating temporal consistency in foraging behaviour (Supplementary Fig. S2). To quantify this consistency, we applied separate PCAs to δ^13^C and δ^15^N dual-tissue data, capturing the shared variability between plasma and red blood cell measurements for each isotope (Supplementary Fig. S3–S4). PC1 scores were standardised into *z*-scores, denoted as δ^15^N_consist_ and δ^13^C_consist_.

Isotopic consistency varied across species and colonies. For nitrogen (δ^15^N_consist_), UA breeding at the southernmost colonies showed predominantly positive *z*-scores (median: SE, 1.3; SW, 1.4), indicating consistently higher δ^15^N values across both tissues relative to other populations. In contrast, the northernmost colonies of both species exhibited negative *z*-scores (ranging from −0.9 to −0.2), indicating consistently lower δ^15^N values across tissues compared with other populations. Carbon isotope consistency (δ^13^C_consist_) displayed similar geographic patterns, with UA from southernmost colonies displaying positive *z*-scores (median: SE, 2.0; SW, 1.6), indicating consistently less negative δ^13^C values, whereas northern populations of both species showed negative *z*-scores (ranging from −0.9 to −0.1), indicating consistently more negative δ^13^C values. Both isotope systems demonstrated consistent north–south gradients, with UL showing relatively similar patterns to northernmost UA.

### 3.3 Conditional segmented regression and predictive risk test

**Fig. 5:**
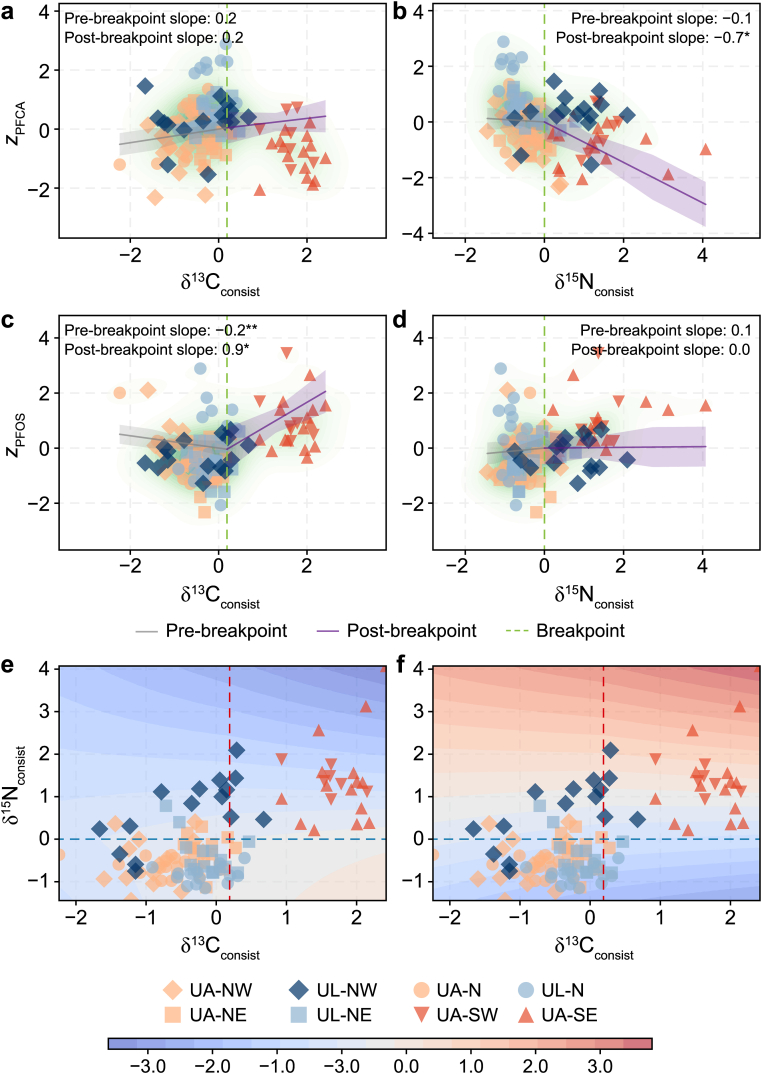
**Bivariate segmented regressions and predictive exposure surface for PFAS variability in guillemots. a**–**d**, Segmented regression relationships between PFAS variability (*z*-scores for long-chain PFCAs [**a**, **b**] and PFOS [**c**, **d**]) and isotopic foraging consistency metrics (δ^13^C [**a**, **c**] and δ^15^N [**b**, **d**]). Vertical dashed green lines indicate identified breakpoints, with pre- and post-breakpoint slopes annotated (asterisks denote significance: ∗*p* < 0.05, ∗∗*p* < 0.01). Shade represents 95% confidence intervals, and underlying green contours represent data density (Kernel Density Estimation). **e**–**f**, Contour plots illustrating two-way interactions between δ^13^C_consist_ and δ^15^N_consist_ for z_PFCA_ (**e**) and z_PFOS_ (**f**). Dashed lines mark identified breakpoints (δ^13^C_consist_ = 0.2, vertical; δ^15^N_consist_ = 0.0, horizontal), delineating four foraging strategy quadrants: Arctic-influenced habitat specialists (δ^13^C_consist_ < 0.2 and δ^15^N_consist_ < 0.0), niche partitioning within Arctic-influenced waters (δ^13^C_consist_ < 0.2 and δ^15^N_consist_ > 0.0), Atlantic-influenced generalists (δ^13^C_consist_ > 0.2 and δ^15^N_consist_ > 0.0), and individuals with high carbon but low nitrogen consistency (δ^13^C_consist_ > 0.2 and δ^15^N_consist_ < 0.0). Colour gradients represent predicted PFAS *z*-scores ranging from −3.0 (blue) to 4.0 (red). Species are distinguished by colours and breeding colonies by marker shapes. UA: *Uria aalge*, common guillemots; UL: *Uria lomvia*, Brünnich's guillemots. Five geographic colonies: southwest (SW), northwest (NW), north (N), northeast (NE), and southeast (SE).

To delineate foraging habitats in isotopic space, we identified isotopic breakpoints (δ^13^C_consist_ = 0.2; δ^15^N_consist_ = 0.0) as biogeochemical transitions, where the coupled carbon–nitrogen values significantly shift. This approach allowed us to distinguish discrete water masses based on their isotopic values and evaluate the corresponding response trajectories of PFCAs and PFOS variability. The segmented regression profiles (Fig. 5a–d) reveal non-linear shifts in variability once these water mass boundaries are surpassed.

For PFCAs, their variability is governed by a nitrogen-mediated attenuation (Fig. 5b). Below the nitrogen breakpoint (δ^15^N_consist_ = 0.0), PFCA variability (z_PFCA_) remains relatively stable (*β*_pre_ = −0.1; Fig. 5b). Crossing this threshold, however, triggers a significant seven-fold acceleration in decline (*β*_post_ = −0.7, *p* < 0.05; Fig. 5b). In contrast, PFOS variability (z_PFOS_) exhibits a slope inversion along the carbon gradient (Fig. 5c). Below the carbon breakpoint (δ^13^C_consist_ = 0.2), PFOS variability (z_PFOS_) shows a significant negative correlation with the carbon values (*β*_pre_ = −0.2, *p* < 0.01; Fig. 5c). Once this threshold is exceeded, the trajectory reverses into a positive slope of considerably greater magnitude (*β*_post_ = 0.9, *p* < 0.05; Fig. 5c), signifying a transition from suppression to elevation in high-carbon regimes. The reconstructed performance surfaces (Fig. 5e and f) reveal two divergent patterns in PFCAs and PFOS variability. z_PFCA_ remains stable in low-nitrogen waters and declines as nitrogen isotope values increase (Fig. 5e). z_PFOS_ remains constrained across low-carbon segments, with a localised increase observed where both carbon and nitrogen values are elevated (Fig. 5f).

Model performance was evaluated by comparing predictive error across training and validation subsets (Table 2). While the absolute variance explained reflects the inherent complexity of PFAS variability, the narrow margin between training and validation error suggests that the identified isotopic thresholds are structurally robust. These breakpoints represent generalised ecological transitions rather than artefacts of the training data. Specifically, 55.0% of total model variance in z_PFCA_ was attributable to the segment effect associated with the nitrogen threshold. z_PFCA_ remained elevated in the Low-Carbon/Low-Nitrogen regime; a systematic decline in relative variability was observed following the nitrogen threshold (Table 2). In contrast, z_PFOS_ showed a significant interaction between carbon and nitrogen segments (δ^13^C_consist_ x δ^15^N_consist_, *p* = 0.03; Table 2). The carbon threshold accounted for 65.7% of the total effect size, with a slope reversal of *β* = 1.2 (*p* < 0.01) observed beyond this threshold. In isolated high-nitrogen conditions, mean z_PFOS_ was −0.4; where both carbon and nitrogen values exceeded their respective thresholds, mean z_PFOS_ increased to 0.8 (full parameter estimates: Supplementary Table S8–S9).

**Table 2:** Model performance and parameter estimate for bivariate segmented regression of PFAS variability across isotopic regimes. ^b^The High-Carbon/Low-Nitrogen regime was excluded from analysis due to insufficient sample size (*n* = 6).

Response variable	Parameter/metric	PFCA (*z*_PFCA_)	PFOS (*z*_PFOS_)
Model performance	*R*^2^_Train_	0.2	0.3
	*R*^2^_Test_	0.1	0.2
	*RMSE*_Train_	0.9	0.8
	*RMSE*_Test_	1.1	1.0
Coefficients	Intercept	0.3	−0.4∗∗
(*p-*value)	δ^13^C_consist_	0.2	−0.2
	δ^13^C_consist_ segment	−0.1	1.2∗∗
	δ^15^N_consist_	−0.1	0.1
	δ^15^N_consist_ segment	−0.6	−0.1
	δ^13^C_consist_ x δ^15^N_consist_	−0.2	0.1∗
Segment means	Low C/Low N (*n* = 65)	0.2	−0.3
	Low C/High N (*n* = 15)	−0.3	−0.4
	High C/High N (*n* = 26)	−0.5	0.8

a. Significance levels: ∗*p* < 0.05, ∗∗*p* < 0.01.

b. Training observations: 84; Testing observations: 22.

c. Variable segment: Interaction with segment (slope change after breakpoint).

d. x: Interaction term between variables.

e. Segment combinations represent different biogeochemical zones identified in the study.

## 4 Discussion and conclusion

PFAS exposure patterns in marine predators reflect the combined influence of environmental exposure pathways and trophic transfer [5,6]. Whereas previous studies have often treated interindividual variability as statistical noise, the present analysis treats it as ecologically informative, revealing aspects of contaminant dynamics that mean-based analyses may overlook [5,6,12]. By using dual-isotope consistency metrics (δ^13^C_consist_ and δ^15^N_consist_ scores), we showed how PFAS variability reflects ecological processes that link foraging behaviour and habitat use to contaminant exposure. Despite the methodological limitations discussed below, this approach offers new insight into the ecological drivers of contaminant dynamics in marine systems [38,69].

### 4.1 PFAS variability as a structured ecological signal

We predicted that (1) PFAS exposure variability would contain structured ecological signals reflecting foraging strategies and oceanographic influences, and (2) UL and UA would exhibit distinct variability profiles, with UL showing more constrained patterns and UA exhibiting greater variability. The results strongly support the first hypothesis, revealing structured variability clusters (Fig. 3) and significant threshold effects at Arctic-Atlantic transitions (Table 2, Fig. 5). However, contrary to our second hypothesis, the relative constraint between species varied by compound class and oceanographic context rather than one species consistently showing more constrained variability.

Three lines of evidence indicated that PFAS variability reflects structured ecological signals rather than statistical noise. First, the spatial organisation of the variability clusters (Fig. 3) provided evidence consistent with exposure regimes being structurally partitioned by the transition between distinct oceanographic water masses. Birds at the northernmost colonies, foraging within cold, Arctic-influenced water masses (indicated by more negative δ^13^C and δ^15^N values; Fig. 4) and assigned predominantly to Cluster 1 (Fig. 3), contrasted strongly with the southernmost populations in warmer, saline Atlantic inflows (more positive δ^13^C and δ^15^N values; Fig. 4), which were mostly assigned to Cluster 2 (elevated PFOS variability; Fig. 3). Where δ^15^N values are interpreted as indicators of relative trophic position, caution is warranted given that isotopic baselines differ between Arctic and Atlantic water masses [70,71]; accordingly, these values are considered indicative of within-region trophic gradients rather than absolute cross-colony trophic comparisons. This geographic pattern is consistent with the hypothesis that contrasting water masses modulate the efficiency with which contaminants move through the food web, though the directionality of this relationship cannot be established from isotopic proxies alone. Specifically, differences in food web structure between water masses, defined here by local prey assemblage composition and food chain length, likely shape variation in the efficiency of PFAS assimilation across colonies. Colonies embedded in more complex or elongated food webs are expected to exhibit more variable PFAS exposure in avian predators relative to those supported by shorter or simpler prey assemblages. In this sense, the oceanographic regime may act as a biogeochemical template that establishes baseline PFAS availability at the base of the local food web, while the ecological structure of that food web—its length, taxonomic composition, and energetic structure—determines the degree and variability of PFAS assimilation into apex predators.

Second, our conditional segmented regression identified isotopic functional breakpoints (δ^13^C_consist_ = 0.2 and δ^15^N_consist_ = 0.0) that are consistent with the transition between distinct oceanographic regimes (Table 2). In the absence of isotopic baselines, these thresholds function as internally derived biogeochemical reference points that distinguish Arctic-influenced water masses from Atlantic-influenced foraging habitats [71]. It is acknowledged that these breakpoints are endogenously determined from the dual-isotope relationship itself; therefore, they are interpreted as identifying where the statistical coupling between isotopic foraging metrics undergoes a structural change, rather than as independent markers of oceanographic boundaries. The oceanographic regime thus appears to define the conditions under which PFAS accumulate, determining whether a given compound class undergoes progressive biomagnification or remains constrained within a relatively narrow concentration range across individuals. Consequently, the spatial organisation observed in the cluster analysis (Fig. 3) is interpreted as a structured outcome of these compound-specific isotopic drivers, where the transition between water masses corresponds to a shift in the prevailing contaminant kinetics.

For PFOS variability, the δ^13^C_consist_ breakpoint corresponded to a discrete reversal in both the direction and magnitude of the PFOS-habitat relationship (Table 2, Fig. 5). Below this carbon threshold, birds with high habitat fidelity to Arctic-influenced habitats (δ^13^C_consist_: −2 to 0) showed a significant negative slope (*β*_pre_ = −0.2), consistent with a constrained accumulation regime in which habitat use limits PFOS variability, the defining characteristic of Cluster 1 (Fig. 3). Above this threshold, as birds shift toward Atlantic-influenced waters, the relationship reversed to positive slope (*β*_post_ = 0.9), consistent with a shift toward biomagnification as the dominant kinetic outcome, producing the concentration (Fig. 2) and variability increases observed across southernmost colonies in Cluster 2 (Fig. 3).

For PFCA variability, trophic position (δ^15^N_consist_) emerged as a candidate modulating factor only within Atlantic-influenced waters (Table 2), suggesting that nitrogen-mediated influence is conditional rather than universal. In Arctic waters, PFCA variability remained statistically decoupled from trophic position; this coupling only becomes apparent following the carbon breakpoint (*β*_post_ = −0.7, *p* < 0.05; Fig. 5b). However, cross-validation substantially qualified this finding: although the nitrogen segment explained 55.0% of training variance, no individual factors reached significance during validation (Table 2). Two non-mutually exclusive interpretations are consistent with this outcome: genuine biochemical attenuation driven by PFCA's shorter half-lives and lower biomagnification potential relative to PFOS [72], or partial model overfitting in the training phase. This compound-specific divergence aligned with the observation established in the first line of evidence: the foraging habitat operated with differing reliability across PFAS classes, and for PFCAs the current model did not yield a generalisable predictive structure.

Taken together, these compound-specific patterns are consistent with a hierarchical structure in which large-scale oceanography (δ^13^C_consist_) constrains the conditions under which micro-scale trophic interactions (δ^15^N_consist_) shape PFAS variability. Within this hierarchy, the oceanographic regime defines the range of possible exposure conditions, while trophic position acts as a secondary modifier, reducing PFCA variability in birds foraging at high trophic levels in Atlantic food webs, and amplifying PFOS variability where biomagnification is most pronounced. The two resulting cluster patterns, constrained PFOS variability in Arctic-influenced birds and elevated PFOS variability in Atlantic-influenced birds, represent repeatable, oceanographically bounded exposure states rather than statistical artefacts.

The biological relevance of these isotopically defined regimes was supported by strong plasma-cell isotope correlations (*r* = 0.7 for both δ^13^C and δ^15^N) and documented high site fidelity during breeding [73]. Although stable isotopes in blood typically integrate dietary information over weeks rather than months or years, this consistency across tissues indicated that individuals were foraging within stable oceanographic settings throughout the breeding season. The association between isotopic patterns and PFAS variability is therefore unlikely to reflect short-term dietary fluctuations; it more plausibly reflects habitat-specific exposure accumulated over a longer period. This makes the identified isotopic thresholds useful indicators of the broader oceanographic conditions shaping PFAS exposure in these predators.

The fine-scale patterns at the NW and N colonies highlight ecological nuances where subtle behavioural shifts result in disproportionate effects on PFAS exposure variability, transcending regional oceanographic regimes predictions. At the NW colony, the interspecies divergence in cluster assignment indicated that individual-level foraging behaviours can deviate from the regional isotopic patterns. Although both species occupy the same generalised habitat (pre-threshold carbon values), and the model predicted similarly low PFOS variability for both, yet only UL adhered to this expectation. UA at this colony were predominantly assigned to Cluster 2 (Fig. 3), exhibiting elevated PFOS variability despite foraging in Arctic-influenced waters. This discrepancy suggests that while the model captures macro-scale habitat use, it cannot fully resolve the fine-scale foraging niches that structure the exposure variability at this site. For UL, high fidelity to glacial fjords and marginal ice zones [58] maintains a profile consistent with the pre-threshold carbon segment and post-threshold nitrogen segments, keeping PFOS variability constrained within Cluster 1 (Fig. 3). In contrast, UA at this site may forage across a broader range of prey through distinct vertical or temporal prey access across the Irminger Current [58], producing a variability pattern diverging from the prevailing Arctic trend.

At the N colony, our analysis encounters another important ecological boundary: fine-scale temporal and vertical heterogeneity. UA were distributed across all three clusters but predominantly grouped in Cluster 1 (low PFOS variability), with only one individual appearing in Cluster 3 (elevated variability across both PFAS classes), whereas UL were more frequently assigned to Cluster 3. This divergence is not explained by the broad δ^13^C gradient, suggesting that temporal or vertical niche differences drive the contrast. Previous tracking and observational data suggest that UL predominantly forages nocturnally within shallow water columns [73], a strategy that provide access to a diverse arrays of vertically migrating prey communities [74,75]. In contrast, the more consistent, deep-diving diurnal behaviour of UA [73], which tend to forage within a more uniform benthic or deep-pelagic food web, results in a more stabilised variability profile. Such temporal and depth-related partitioning may have increased exposure heterogeneity for UL relative to the more consistent deep-diving behaviour of UA.

At the NE colony, both species were predominantly assigned to Cluster 1 (Fig. 3). This convergence is consistent with the absence of niche partitioning between species reported for this site [58], and with the significant negative segmented slope below the carbon threshold (Table 2), which predicted low and consistent PFOS variability for birds foraging in Arctic-influenced waters. The NE pattern therefore revealed how shared access to similar prey in an oceanographically uniform setting can reduce interindividual variability in PFAS exposure.

At the two southernmost colonies, UA populations were predominantly grouped in Cluster 2 (Fig. 3), with elevated PFOS variability and lower PFCA variability. This is consistent with the positive PFOS slope above the carbon threshold (Fig. 5d): birds foraging in Atlantic-influenced food webs at elevated trophic positions showed greater between-individual variation in PFOS concentrations (Fig. 2), which the segmented regression associated with increasing δ^13^C_consist_ values in this oceanographic regime. This alignment between cluster distributions and segmented regression slopes suggested that compound-specific accumulation mechanisms are at work [72,76]. For PFOS, higher concentrations in Atlantic-influenced birds may amplify between-individual differences that would otherwise be below detection. For PFCAs, competitive binding to serum albumin, in which PFOS may displace PFCAs from a limited number of binding sites, could reduce detectable interindividual variation in PFCA concentrations regardless of prey diversity. These mechanisms remain speculative and require experimental validation, but they offer a plausible biochemical basis for the divergent variability patterns of the two compound classes.

In Arctic-influenced colonies, where PFAS concentrations are lower and biomagnification appears to be less intense, the contrast is reversed. Lower overall PFAS concentrations likely constrain interindividual variation, while the absence of strong competitive binding permits ecological factors, such as prey diversity and habitat use, to be reflected in PFCA concentrations more directly. These observations suggest that the detectability of ecological variation in PFAS tissue concentrations is itself contingent on concentration regime: at lower concentrations, variability in PFCA patterns reflects individual foraging differences, whereas at higher concentrations, PFOS variability becomes more sensitive to trophic differences and PFCA variability is potentially suppressed by protein-binding competition.

### 4.2 Methodological considerations

We used temporal consistency in isotopic values (δ^13^C_consist_ and δ^15^N_consist_ scores) to characterise foraging behaviour and interpret PFAS variability across oceanographic gradients. While these consistency metrics provide insight into foraging during the breeding season, several methodological limitations should be considered when interpreting the results.

A key limitation is the temporal mismatch between isotope turnover rates (weeks to months) and PFAS persistence, which can span years for some compounds. Although isotopes in both blood fractions reflect recent foraging behaviour, a portion of the measured PFAS burden likely accumulated prior to the breeding season. This issue is particularly relevant for legacy compounds such as PFOS, which has a biological half-life of several years in seabirds [77]. Additionally, isotopic baselines vary substantially across oceanographic regions, seasons, and spatial scales [70,78]. In the absence of direct baseline measurements across the study area, it is not possible to fully disentangle the trophic effects of habitat from baseline-driven isotopic variation. For example, elevated δ^15^N values may reflect shifts in baseline nitrogen sources rather than actual trophic positions [79].

Physiological factors unrelated to diet may also influence both isotopic values and PFAS accumulation. Individual variation in metabolic rate, growth, reproductive status, or detoxification capacity could independently affect isotopic values and PFAS retention, potentially generating correlations that do not directly reflect ecological processes. Finally, the correlative nature of this study precludes causal inference between isotopic patterns and PFAS variability. While the 2018 breeding-season data reveal consistent habitat-associated patterns, experimental validation is required to elucidate the mechanistic links between foraging ecology and PFAS accumulation.

Several methodological advances could address these limitations. Isotope analysis of tissues formed outside the breeding season, along with compound-specific isotope analyses, may provide more detailed insights into foraging behaviour and physiological processes than bulk stable isotope analyses [25,[80], [81], [82], [83], [84], [85]]. Additionally, explicit baseline corrections across oceanographic regions would improve comparability between Arctic and Atlantic systems [85].

### 4.3 Conclusion and implications

PFAS exposure variability in the studied guillemots is shaped by oceanographic gradients, with the Arctic–Atlantic transition marking the most important threshold: PFOS variability is constrained and consistent in Arctic-influenced birds and substantially higher in Atlantic-influenced birds, reflecting the more intense biomagnification associated with warmer, more productive Atlantic food webs. Long-chain PFCAs variability responds more weakly to trophic position, and only within Atlantic-influenced waters, consistent with the shorter biological half-lives and lower protein-binding affinity of PFCAs relative to PFOS. These compound-specific responses highlight the importance of physicochemical properties in governing how ecological information is expressed in tissue concentrations.

The results indicate that isotope values do not predict PFAS variability uniformly across all settings. Whether trophic position is informative depends on the prevailing oceanographic context, and analyses that apply linear models across the full dataset without accounting for this conditionality will tend to obscure important variation. An approach that identifies habitat-associated threshold effects, as applied here, is better suited to capturing these non-linear relationships. As climate change continues to alter the extent and distribution of Arctic and Atlantic water masses around Iceland and throughout subpolar seas, the boundary between constrained-variability Arctic exposure regimes and elevated-variability Atlantic ones is likely to shift. Understanding how these thresholds move, and how individual seabird foraging strategies respond, will be important for anticipating future changes in contaminant exposure in high-latitude marine predators.

## CRediT authorship contribution statement

**Rui Shen:** Writing – review & editing, Writing – original draft, Visualization, Validation, Software, Methodology, Formal analysis, Data curation, Conceptualization. **Ralf Ebinghaus:** Funding acquisition, Conceptualization. **Daniel Giddings Vassão:** Writing – review & editing, Validation, Methodology. **Norman Ratcliffe:** Writing – review & editing, Funding acquisition, Conceptualization. **Thomas Larsen:** Writing – review & editing, Funding acquisition, Formal analysis, Conceptualization.

## Declaration of competing interest

The authors declare that they have no known competing financial interests or personal relationships that could have appeared to influence the work reported in this paper.
