# Supplementary material for: Oceanographic regime and foraging behaviour structure compound-specific PFAS variability in arctic-atlantic guillemots
Source: Environ Sci Ecotechnol. 2026 May 14;31:100707. doi: 10.1016/j.ese.2026.100707 (PMC13223959; doi:10.1016/j.ese.2026.100707)
Supplement: Multimedia component 1 [file mmc1.docx]

Supplementary Information

**Oceanographic regime and foraging behaviour structure compound-specific PFAS variability in Arctic-Atlantic guillemots**

Rui Shen^a^*, Ralf Ebinghaus^b^, Daniel Giddings Vassão^a,c^, Norman Ratcliffe^d^, Thomas Larsen^a,e^

^a^Max Planck Institute of Geoanthropology, Jena 07745, Germany

^b^Institute of Coastal Environmental Chemistry, Helmholtz Zentrum hereon, Geesthacht 21502, Germany

^c^Max Planck Institute for Chemical Ecology, Jena 07745, Germany

^d^British Antarctic Survey, Natural Environment Research Council, Cambridge CB3 0ET, UK

^e^Institute for Prehistoric and Protohistoric Archaeology, Christian-Albrechts-Universität zu Kiel, Kiel 24118, Germany

*Corresponding author: [rshen@gea.mpg.de](mailto:rshen@gea.mpg.de)

**Summary Information:**

- **Total Pages:** 18
- **Supplementary Figures:** S1–S6
- **Supplementary Tables:** S1–S9

# Supplementary Methods

## Sample Collection

Following centrifugation, plasma aliquots were transferred into borosilicate‑glass Petri dishes that had been rinsed with deionised water and pre‑combusted at 500°C for 30 minutes to minimise PFAS sorption [1]. Samples were covered with pre‑combusted glass lids and placed in a polyethylene vacuum desiccator for four to six days of ambient temperature drying to exclude airborne contaminants. Once dry, dishes were sealed with Parafilm, double‑bagged in PFAS‑free polyethylene zip‑bags and stored at room temperature until extraction.

## Target Compounds & Standards

**Table S1.** PFAS analytes. Chemical information of target per‑ and polyfluoroalkyl substances (PFAS) analysed in this study. The compounds are categorized into three major classes: carboxylic acids (C5-C13), sulfonic acids (C4, C6, C8, & C10), and ether (HFPO‑DA). All analytical standards were obtained from Wellington Laboratories with certified purity >98%. The PFC‑MXA mixture was prepared at 2.0 µg/mL ± 5%, while the HFPO-DA individual standard was prepared at 50 ± 2.5 µg/mL.

| **Class** | **Acronym** | **Chemical Name** | **CAS No.** | **Standard Information** |
| --- | --- | --- | --- | --- |
| Carboxylic Acids | PFPeA | perfluoro*-n-*pentanoic acid | 2706-90-3 | PFC-MXA mixture, |
|  | PFHxA | perfluoro*-n-*hexanoic acid | 307-24-4 | Wellington Laboratories, |
|  | PFHpA | perfluoro*-n-*heptanoic acid | 375-85-9 | 2.0 µg/mL ± 5%, |
|  | PFOA | perfluoro*-n-*octanoic acid | 335-67-1 | >98% |
|  | PFNA | perfluoro*-n-*nonanoic acid | 375-95-1 |  |
|  | PFDA | perfluoro*-n-*decanoic acid | 335-76-2 |  |
|  | PFUnDA | perfluoro*-n-*undecanoic acid | 2058-94-8 |  |
|  | PFDoDA | perfluoro*-n-*dodecanoic acid | 307-55-1 |  |
|  | PFTrDA | perfluoro*-n-*tridecanoic acid | 72629-94-8 |  |
| Sulfonic Acids | PFBS | potassium perfluoro*-n-*butanesulfonate | 375-73-5 |  |
|  | PFHxS | sodium perfluoro-*n-*hexanesulfonate | 355-46-4 |  |
|  | PFOS | sodium perfluoro*-n-*octanesulfonate | 1763-23-1 |  |
|  | PFDS | sodium perfluoro*-n-*decanesulfonate | 335-77-3 |  |
| Ether | HFPO-DA | 2,3,3,3-tetrafluoro-2-(1,1,2,2,3,3,3-heptafluoropropoxy)propanoic acid | 13252-13-6 | Individual standard, Wellington Laboratories, |
|  |  |  |  | (50 ± 2.5) µg/mL, |
|  |  |  |  | >98% |

**Table S2.** List of solvents and reagents used in the analytical procedure. All chemicals were of high analytical grade suitable for LC‑MS analysis or equivalent high-purity grade. Ultrapure water was produced in-house using a Milli‑Q water purification system. The reagent grade, purity specifications, and suppliers are provided to ensure reproducibility of the analytical method.

| **Chemical** | **Grade/Purity** | **Supplier** |
| --- | --- | --- |
| Methanol | LC-MS grade (LiChrosolv) | Merck |
| Ultrapure water | 18.2 MΩ·cm at 25 °C | Milli-Q Integral 5, Merck |
| Acetic acid | ≥99.8%, LC-MS grade | Honeywell Fluka |
| Ammonium acetate | LC-MS grade | Honeywell Fluka |
| Ammonia solution | 25%, Suprapur | Merck |

## Sample Preparation

PFAS extraction from plasma samples was performed using a modified quaternary ammonium salt‑based ion-pairing method [2,3]. Prior to extraction, plasma samples were fortified with mass‑labelled internal standards and buffered with sodium carbonate (1 mL, 0.5 M, pH 10.0). Tetrabutylammonium hydrogen sulphate (2 mL, 0.5 M) was added as the ion‑pairing agent.

The analytes were extracted three times with ethyl acetate (5 mL per extraction) using ultrasonication (20 min, room temperature) followed by centrifugation (3000 rpm, 15 min, 20°C). The combined organic extracts were concentrated under a gentle nitrogen stream at 40°C and reconstituted in methanol: water (1:1 v/v, 250 µL). Final extracts were filtered through a 0.2 µm polypropylene membrane filter prior to analysis.

## Instrumental Analysis

**Table S3.** Detailed instrumental parameters for the HPLC-MS/MS analysis of PFAS. The chromatographic separation was performed using an Agilent 1100 HPLC system coupled to an AB Sciex API 4000 triple quadrupole mass spectrometer. The method utilized a reverse-phase C18 column with gradient elution using ammonium acetate buffer and methanol-based mobile phases. Mass spectrometric detection was performed using electrospray ionization in negative mode with multiple reaction monitoring (MRM).

| **System** | **Parameter** | **Specification** |
| --- | --- | --- |
| **HPLC** | System | HP 1100, Agilent Technologies |
|  | Analytical Column | Synergi Fusion-RP C18, 150 × 2 mm, 4 µm, 80 Å (Phenomenex) |
|  | Guard Column | SecurityGuard C18, 4 × 2 mm (Phenomenex) |
|  | Mobile Phase A | 2 mM ammonium acetate in water |
|  | Mobile Phase B | 0.05% acetic acid in methanol |
|  | Flow Rate | 0.2 mL/min |
|  | Injection Volume | 10 µL |
|  | Column Temperature | 30 °C |
|  | Run Rime | 30 min |
| **MS/MS** | System | API 4000 triple quadrupole, AB Sciex |
|  | Source | Turbo V ESI, negative mode |
|  | Ion Spray Voltage | -4500 V |
|  | Source Temperature | 400 °C |
|  | Gases (N_2_) | Nebulizer: 4.2 bar |
|  |  | Heater: 2.8 bar |
|  |  | Curtain: 1.0 bar |
|  |  | Collision: 0.6 bar |
|  | Scan Type | MRM |

**Table S4.** LC-MS/MS parameters for the analysis of per- and polyfluoroalkyl substances (PFAS) and their corresponding internal standards. The table presents molecular formulas of detected ions, quantifier and qualifier transitions (m/z), and matched internal standards for each target analyte. The compounds are categorized into native PFCAs (C5‑C13), PFSAs (C4, C6, C8 & C10), HFPO-DA, and isotope‑labelled internal standards. Quantifier ion transitions (marked with asterisk) were used for quantitation, while qualifier transitions were monitored for confirmation where available.

| Type | Compound | Molecular Formula | Quantifier Transition (m/z) | Qualifier Transition (m/z) | Internal Standard |
| --- | --- | --- | --- | --- | --- |
| Native PFCAs | PFPeA | [C5F9O2]- | 263 > 219* | - | ^13^C_2_-PFHxA |
|  | PFHxA | [C6F11O2]- | 313 > 269* | 313 > 119 | ^13^C_2_-PFHxA |
|  | PFHpA | [C7F13O2]- | 363 > 169* | 363 > 319 | ^13^C_4_-PFOA |
|  | PFOA | [C8F15O2]- | 413 > 369* | 413 > 169 | ^13^C_4_-PFOA |
|  | PFNA | [C9F17O2]- | 463 > 419* | 463 > 219 | ^13^C_5_-PFNA |
|  | PFDA | [C10F19O2]- | 513 > 469* | 513 > 219 | ^13^C_2_-PFDA |
|  | PFUnDA | [C11F21O2]- | 563 > 519* | 563 > 169 | ^13^C_2_-PFUnDA |
|  | PFDoDA | [C12F23O2]- | 613 > 569* | 613 > 169 | ^13^C_2_-PFDoDA |
|  | PFTrDA | [C13F25O2]- | 663 > 619* | 663 > 169 | ^13^C_2_-PFDoDA |
| Native PFSAs | PFBS | [C4F9O3S]- | 299 > 80* | 299 > 99 | ^18^O_2_-PFHxS |
|  | PFHxS | [C6F13O3S]- | 399 > 80* | 399 > 99 | ^18^O_2_-PFHxS |
|  | PFOS | [C8F17O3S]- | 499 > 80* | 499 > 99 | ^13^C_4_-PFOS |
|  | PFDS | [C10F21O3S]- | 599 > 80* | 599 > 99 | ^13^C_4_-PFOS |
| Native Ether | HFPO-DA | [C6F11O3]- | 329 > 285 | - | ^13^C_3‑_HFPO‑DA |
| Internal Standards | ^13^C_2_-PFHxA | [13C2C4F11O2]- | 315 > 270* | 315 > 120 | - |
|  | ^13^C_4_-PFOA | [13C4C4F15O2]- | 417 > 372* | 417 > 169 | - |
|  | ^13^C_8_-PFOA | [13C8F15O2]- | 421 > 376* | 421 > 172 | - |
|  | ^13^C_5_-PFNA | [13C5C4F17O2]- | 468 > 423* | 468 > 223 | - |
|  | ^18^O_2_-PFHxS | [C6F13O2OS]- | 403 > 84* | 403 > 103 | - |
|  | ^13^C_4_-PFOS | [13C4C4F17O3S]- | 503 > 80* | 503 > 99 | - |
|  | ^13^C_3‑_HFPO‑DA | [13C3C3F11O3]- | 332 > 287* | 332 > 169 | - |
| *Quantifier ion transition | | | | | |

## Quality Assurance/Quality Control

Background PFAS levels were evaluated through procedural blanks analysed with each batch of 10 samples. The Limit of Blank (LoB) was calculated as[4]:

$$LoB=mean(blank)+1.645(SDblank)$$

Sample concentrations above LoB were considered detectable and were blank corrected by subtracting the mean blank values. Values below LoB were reported as not detected. Recovery rates for all target compounds and their mass-labelled internal standards are summarized in Table A5.

**Table S5.** Mean percent recoveries and standard deviations (SD) for isotope-labelled per- and polyfluoroalkyl substances (PFAS) internal standards.

| **Standard** | **Mean** | **SD** |
| --- | --- | --- |
| ^13^C_2_-PFHxA | 78% | 10% |
| ^13^C_4_-PFOA | 88% | 11% |
| ^13^C_5_-PFNA | 87% | 11% |
| ^13^C_2_-PFDA | 86% | 17% |
| ^13^C_2_-PFUnDA | 87% | 22% |
| ^13^C_2_-PFDoDA | 102% | 17% |
| ^18^O_2_-PFHxS | 93% | 12% |
| ^13^C_4_-PFOS | 102% | 14% |
| ^13^C_4_-PFDS | 94% | 13% |
| ^13^C_3‑_HFPO‑DA | 131% | 80% |

Calibration curves showed good linearity (*R*² > 0.995) over the concentration range of 0.0 - 100 pg/µL. Method detection limits (MDL) were determined based on signal-to-noise ratio calculations following the guidelines outlined in Agilent's technical report on mass spectrometry detection limits [5]. The calculated MDL, LoB and mean blank values for each target compound are detailed in Table A6.

**Table S6.** Method performance parameters for target PFAS

| **Class** | **Acronym** | **MDL [ng/mL]** | **LoB [ng/mL]** | **Blanks [ng/mL]** |
| --- | --- | --- | --- | --- |
| Carboxylic | PFPeA | 0.06 | 0.23 | 0.22 |
| Acids | PFHxA | 0.06 | 0.33 | 0.30 |
|  | PFHpA | 0.06 | < 0.06 | < 0.06 |
|  | PFOA | 0.06 | 0.11 | 0.13 |
|  | PFNA | 0.06 | < 0.06 | < 0.06 |
|  | PFDA | 0.07 | < 0.07 | < 0.07 |
|  | PFUnDA | 0.05 | < 0.05 | < 0.05 |
|  | PFDoDA | 0.05 | < 0.05 | < 0.05 |
|  | PFTrDA | 0.06 | < 0.06 | < 0.06 |
| Sulfonic | PFBS | 0.06 | < 0.06 | < 0.06 |
| Acids | PFHxS | 0.06 | < 0.06 | < 0.06 |
|  | PFOS | 0.05 | 0.34 | 0.28 |
|  | PFDS | 0.06 | < 0.06 | < 0.06 |
| Ether | HFPO-DA | 0.06 | < 0.06 | < 0.06 |

# Supplementary Results

## PFAS Analysis

### PCA


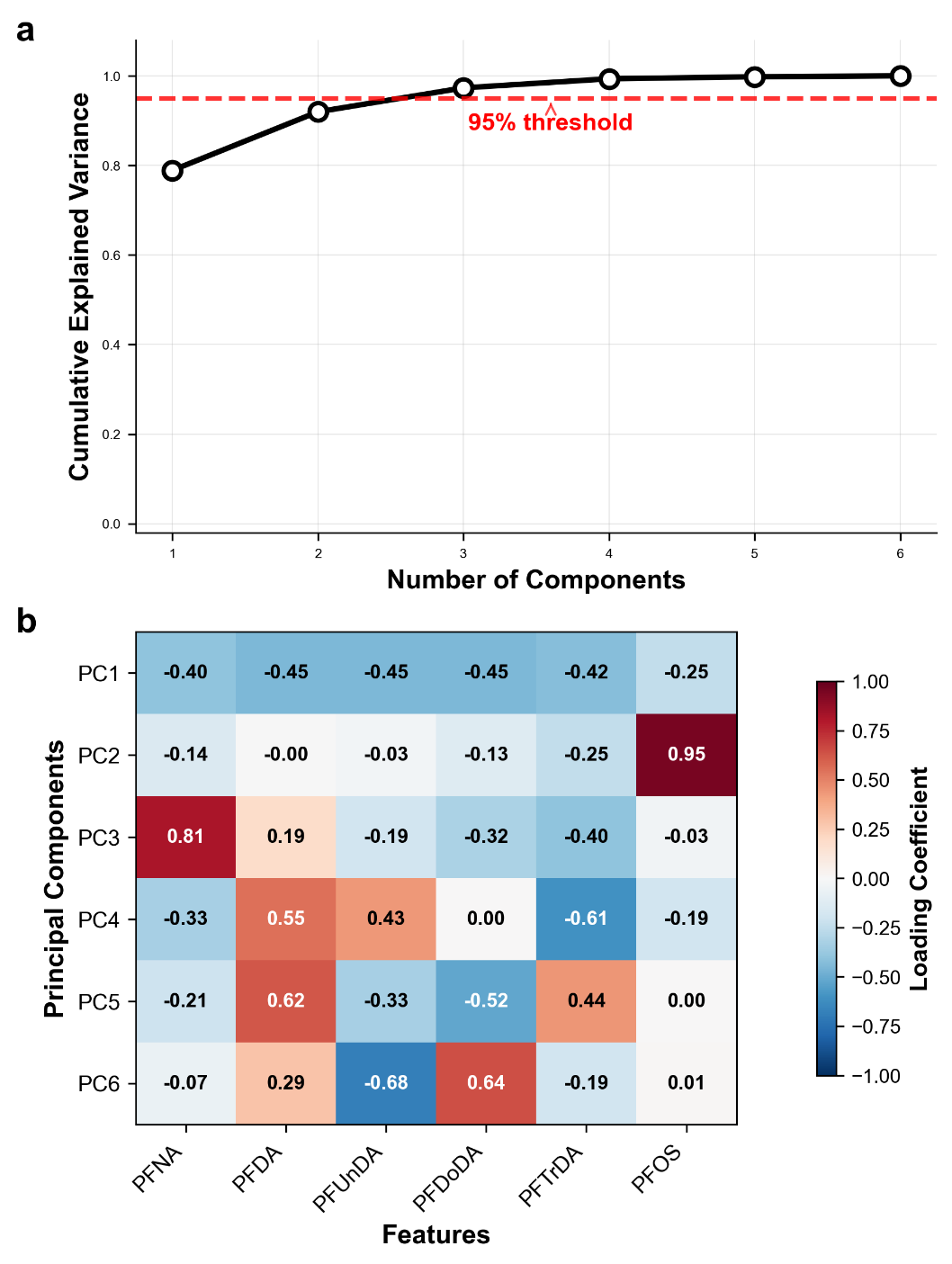


**Figure S1.** Principal Component Analysis (PCA) visualization of the PFAS dataset. (a) Explained variance ratio across principal components, showing the proportion of variance captured by each component. (b) Heatmap depicting feature contributions to the first six principal components, where colour intensity represents loading strength and direction.

## Stable Isotope Analysis

### Descriptives of SIR

**Table S7.** Statistical summary of stable isotope values (δ¹⁵N and δ¹³C) in cell and plasma samples from UA (*n* = 67) and UL (*n* = 45) groups.

| **Species** | **Colony** | **δ^15^N_cell_** | | **δ^13^C_cell_** | | **δ^15^N_plasma_** | | **δ^13^C_plasma_** | |
| --- | --- | --- | --- | --- | --- | --- | --- | --- | --- |
|  |  | **Median** | **IQR** | **Median** | **IQR** | **Median** | **IQR** | **Median** | **IQR** |
| UA | N | 11.8 | 0.3 | -20.3 | 0.1 | 11.9 | 0.2 | -21.8 | 0.4 |
| UA | NE | 12.2 | 0.3 | -20.0 | 0.2 | 11.9 | 0.4 | -21.4 | 0.3 |
| UA | NW | 11.8 | 0.4 | -20.4 | 0.2 | 12.0 | 0.6 | -21.8 | 0.7 |
| UA | SE | 13.1 | 0.7 | -19.2 | 0.4 | 13.1 | 0.4 | -19.8 | 0.2 |
| UA | SW | 13.2 | 0.3 | -19.3 | 0.2 | 13.2 | 0.4 | -19.9 | 0.4 |
| UL | N | 11.8 | 0.2 | -20.2 | 0.1 | 11.6 | 0.3 | -21.3 | 0.4 |
| UL | NE | 12.1 | 0.2 | -20.0 | 0.1 | 11.7 | 0.4 | -21.3 | 0.5 |
| UL | NW | 12.8 | 0.8 | -19.9 | 0.7 | 12.6 | 0.7 | -21.6 | 0.8 |

### Correlation Analysis


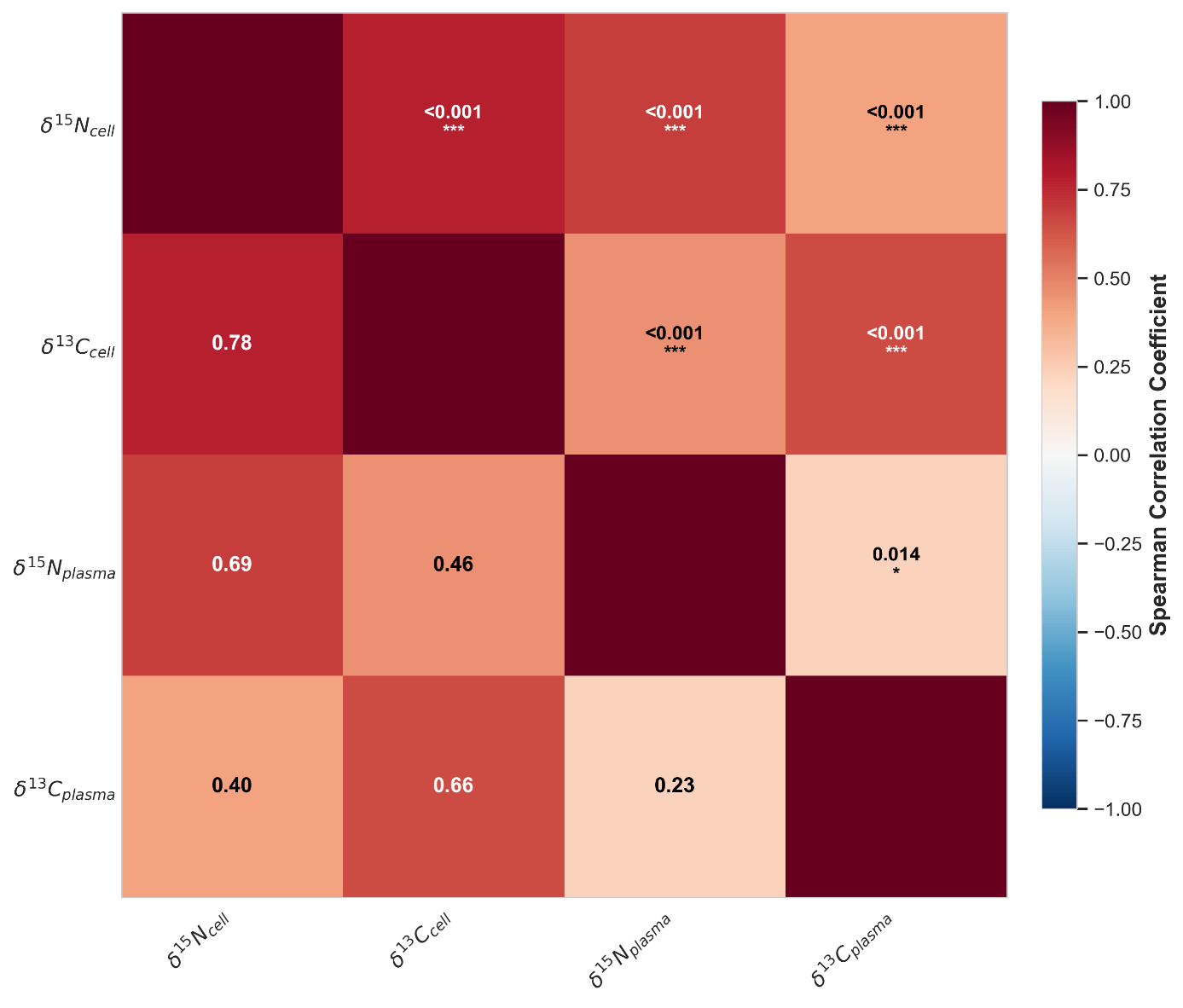


**Figure S2.** Spearman correlation heatmap of stable isotope values across tissue types. Correlation matrix illustrates the relationships between carbon (δ¹³C) and nitrogen (δ¹⁵N) isotope values in cell and plasma tissues. Colour intensity represents correlation strength, with red indicating positive correlations and blue indicating negative correlations. Asterisks denote statistical significance levels: *** *p* < 0.001, ** *p*< 0.01, * *p* < 0.05, ns *p* ≥ 0.05. Values within each cell represent the Spearman correlation coefficient.

### PCA


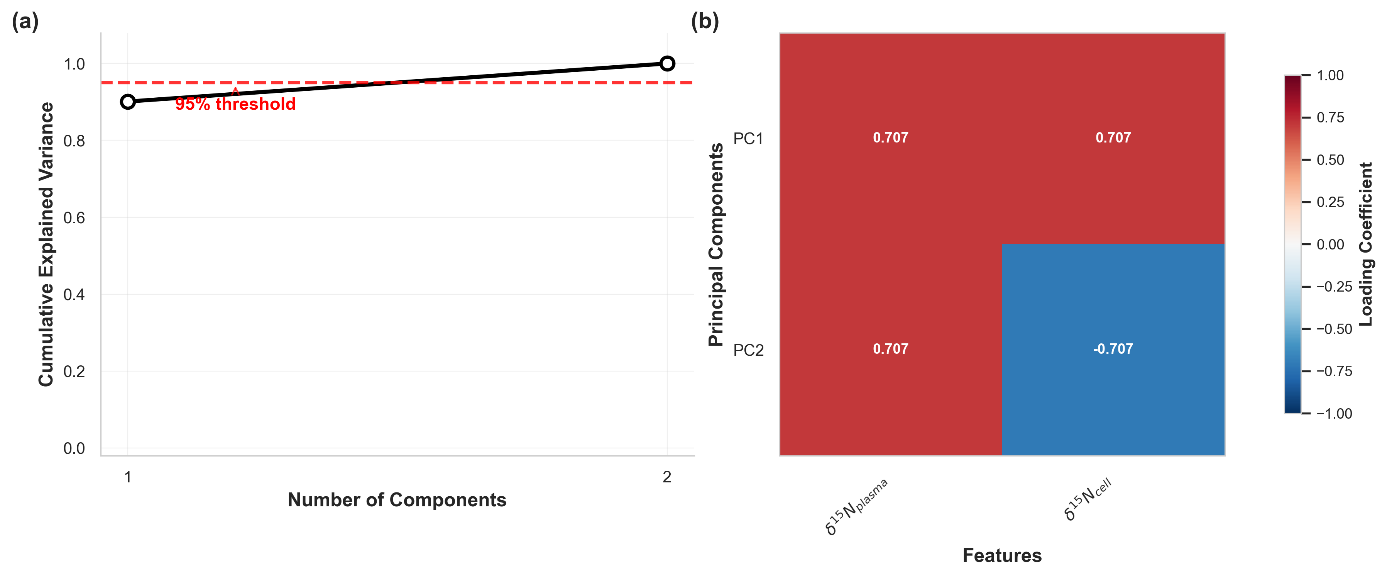


**Figure S3.** Principal Component Analysis of δ^15^N in plasma and cells. **(a)** Scree plot showing cumulative explained variance by principal components. The red dashed line indicates the 95% variance threshold. **(b)** Feature contributions (loadings) to principal components PC1 and PC2. Loading coefficients range from -1 to +1, with red indicating positive loadings and blue indicating negative loadings. PC1 explains 90.1% of the total variance, while PC2 explains the remaining 9.9%.


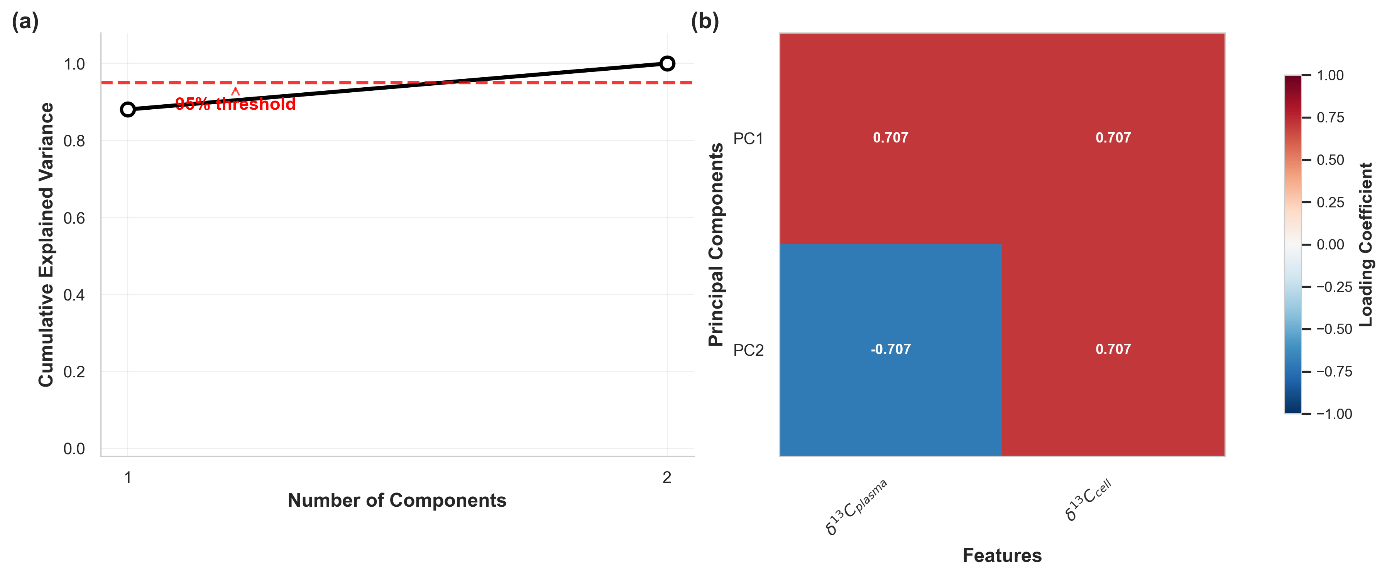


**Figure S4.** Principal Component Analysis of δ^13^C in plasma and cells. **(a)** Scree plot showing cumulative explained variance by principal components. The red dashed line indicates the 95% variance threshold. **(b)** Feature contributions (loadings) to principal components PC1 and PC2. Loading coefficients range from -1 to +1, with red indicating positive loadings and blue indicating negative loadings. PC1 explains 88.1% of the total variance, while PC2 explains the remaining 11.9%.

## Bivariate Threshold Detection

A continuous piecewise (segmented) regression was used to identify ecological thresholds (breakpoints). The model characterises the relationship between isotopes using two linear segments constrained to meet at a breakpoint (*ψ*). Given the standardised nature of the data, the intercept was fixed at zero:

$$y=\left\{ \begin{aligned} \beta_{1}x, &x<\psi\\ \beta_{1}x+\beta_{2}(x-\psi) , &x\geq\psi\end{aligned} \right.$$

Where *β_1_* and *β_2_* are the slopes before and after the breakpoint, respectively.

Breakpoints were determined by minimizing the Mean Squared Error through a three-stage process:

- Initialisation: A heuristic search across the 25th, 50th, and 75th percentiles provided the initial parameter seeds.
- Refinement: Parameter estimates were finalized using dual optimization solvers: L-BFGS-B (gradient-based) and Nelder-Mead (simplex-based) to ensure global convergence.
- Constraints: To maintain statistical power for slope estimation, breakpoints were restricted to the inter-decile range (10th–90th percentiles).

Thresholds were calculated independently for each isotopic axis:

1. Vertical Threshold (Carbon): Derived by regressing δ^15^N on δ^13^C to find the carbon breakpoint (BP_x_).
2. Horizontal Threshold (Nitrogen): Derived via inverse regression of δ^13^C on δ^15^N to find the nitrogen breakpoint (BP_y_).

The intersection of BP_x_ and BP_y_ partitions the niche space into four quadrants.

## Conditional Segmented Regression

The model was fitted using Ordinary Least Squares, with the objective function minimising the sum of squared residuals across all segments. The response variable z_PFAS_ is modelled as a function of two predictors (δ^15^N_consist_, δ^13^C_consist_) with estimated breakpoints *ψ* (BP_x_, BP_y_). The localised linear segments are defined by the identity function *I*(·), which takes the value 1 if the condition is met and 0 otherwise.

The structural equation for the segments in panels **a–d** is:

$$y=\beta_{pre}x_{1}+\beta_{post}[\left( x-\psi\right) \cdot I\left( x>\psi\right)]$$

Where:

- *β_pre_* is the slope before the threshold.
- *β_post_* is the difference in slope after the threshold.

To account for potential heteroscedasticity across ecological thresholds, we implemented a Segment-Aware Residual Analysis. Confidence Intervals were calculated independently for pre- and post-breakpoint regimes. To ensure robustness against ecological outliers, the standard deviation equivalent (σ_seg_) was derived from the Median Absolute Deviation:

*σ_seg_* = 1.4826 median(|r_i_ - median(r)|)

Where r_i_ represents the residuals within a specific segment.

## Predictive Risk Analysis

The contour plots in panels e–f represent a Bivariate Response Surface. This surface is generated by projecting the fitted model across a continuous grid of δ^15^N_consist_, δ^13^C_consist_.

To prioritize broad trends over local stochasticity in the bivariate surface, the predicted response surface (z_PFAS_) was processed using a Gaussian smoothing filter. This is expressed mathematically as the convolution of the raw predicted grid with a bivariate Gaussian kernel (*G*):

z_smooth_ = z_PFAS_ * G(σ)

In this study, a smoothing width of σ = 1.0 was applied. This value was selected to suppress high-frequency noise resulting from individual biological variation while preserving the structural integrity of the identified isotopic thresholds and the overall topography of the exposure surface.


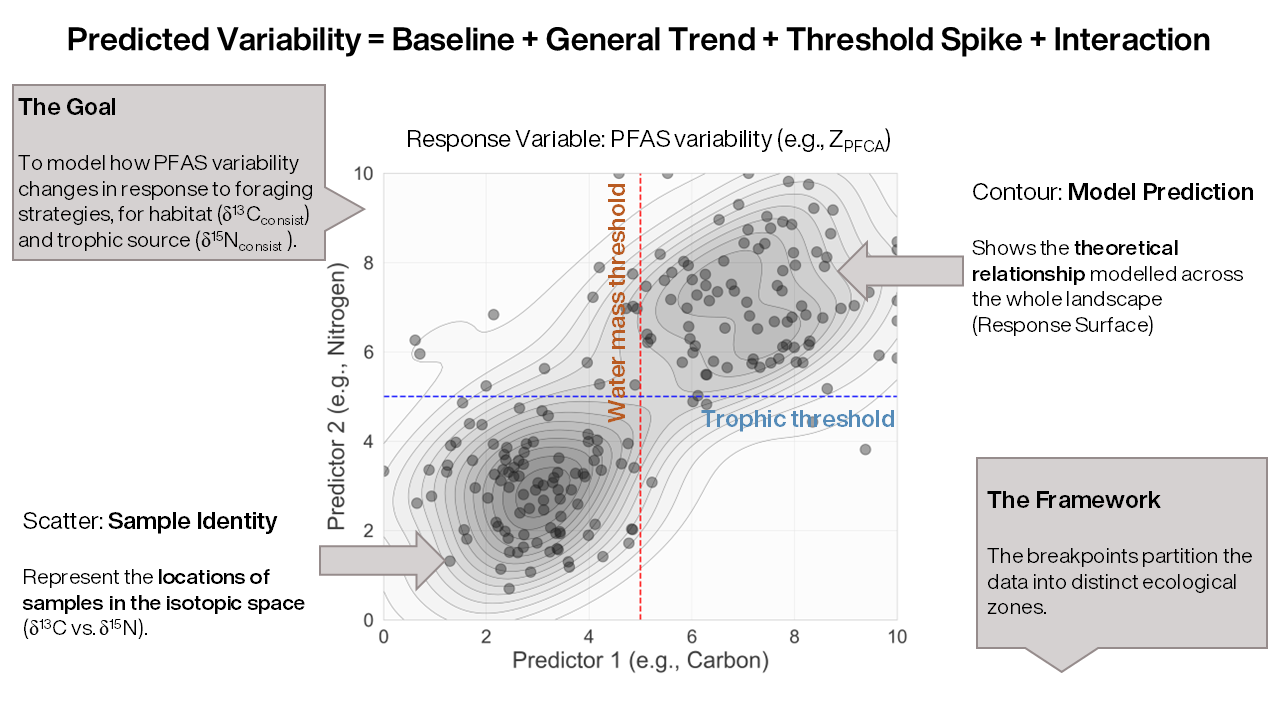


**Figure S5.** Schematic representation of the bivariate segmented response surface. This conceptual diagram illustrates the integration of sample identity (scatter points) with the theoretical response surface (contours). The model partitions the isotopic landscape of guillemot foraging consistency into four ecological zones using statistically identified thresholds (water mass and trophic breakpoints). The resulting surface predicts PFAS variability as a composite of baseline effects, linear trends, and threshold-induced slope shifts.

## Train/Test Validation


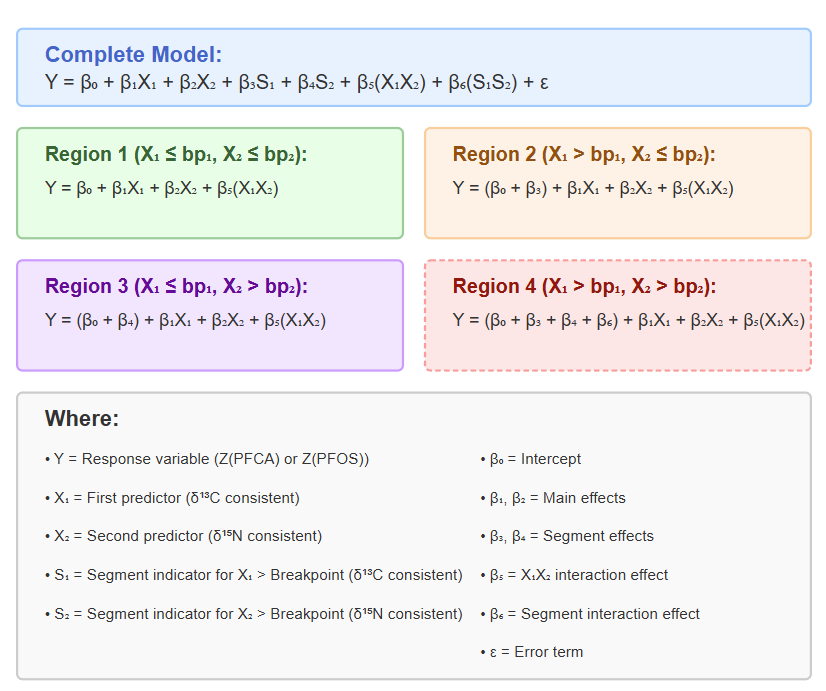


**Figure S6.** Segmented regression model with dual breakpoints for isotope values (δ¹³C and δ¹⁵N). Due to small sample size, Region 4 was excluded from the analysis; the model was implemented using only Regions 1, 2, and 3 combined.

**Table S8.** Bivariate segmented regression results (PFCA)

| **Component** | **Parameter** | **Value** | **SE** | ***t*** | ***p*** | **Training** | **Testing** | **% Effect** | **n** | **Mean** | **SD** | **Min** | **Max** |
| --- | --- | --- | --- | --- | --- | --- | --- | --- | --- | --- | --- | --- | --- |
| **Model Info** | Response Variable | Z_PFCA_ | - | - | - | - | - | - | - | - | - | - | - |
|  | Predictor 1 | δ^13^C_consist_ | - | - | - | - | - | - | - | - | - | - | - |
|  | Predictor 2 | δ^15^N_consist_ | - | - | - | - | - | - | - | - | - | - | - |
|  | Training Observations | 89 | - | - | - | - | - | - | - | - | - | - | - |
|  | Testing Observations | 23 | - | - | - | - | - | - | - | - | - | - | - |
| **Performance** | *R*² | - | - | - | - | 0.19 | 0.03 | - | - | - | - | - | - |
|  | MSE | - | - | - | - | 0.78 | 1.12 | - | - | - | - | - | - |
|  | RMSE | - | - | - | - | 0.88 | 1.06 | - | - | - | - | - | - |
| **Breakpoints** | δ^13^C_consist_ | 0.19 | - | - | - | - | - | - | - | - | - | - | - |
|  | δ^13^C_consist_ Below | 80 (71.4%) | - | - | - | - | - | - | - | - | - | - | - |
|  | δ^13^C_consist_ Above | 32 (28.6%) | - | - | - | - | - | - | - | - | - | - | - |
|  | δ^15^N_consist_ | 0 | - | - | - | - | - | - | - | - | - | - | - |
|  | δ^15^N_consist_ Below | 71 (63.4%) | - | - | - | - | - | - | - | - | - | - | - |
|  | δ^15^N_consist_ Above | 41 (36.6%) | - | - | - | - | - | - | - | - | - | - | - |
| **Coefficients** | Intercept | 0.54 | - | - | - | - | - | - | - | - | - | - | - |
|  | δ^13^C_consist_ | 0.34 | 0 | 1.96 | 0.05 | - | - | 13.18 | - | - | - | - | - |
|  | δ^13^C_consist_ segment | 0.27 | 1 | 0.5 | 0.62 | - | - | 10.5 | - | - | - | - | - |
|  | δ^15^N_consist_ | 0.16 | 0 | 0.98 | 0.33 | - | - | 6.11 | - | - | - | - | - |
|  | δ^15^N_consist_ segment | -0.74 | 0 | -2.4 | **0.02*** | - | - | 28.32 | - | - | - | - | - |
|  | δ^13^C_consist_ × δ^15^N_consist_ interaction | -0.22 | 0 | -2.4 | **0.02*** | - | - | 8.44 | - | - | - | - | - |
|  | Segment interaction | -0.87 | 1 | -1.2 | 0.23 | - | - | 33.45 | - | - | - | - | - |
| **Segment Combinations** | Low δ^13^C_consist_, Low δ^15^N_consist_ | - | - | - | - | - | - | - | 65 | 0.21 | 1 | -1.5 | 2.9 |
|  | Low δ^13^C_consist_, High δ^15^N_consist_ | - | - | - | - | - | - | - | 15 | -0.32 | 1 | -2.3 | 1.5 |
|  | High δ^13^C_consist_, Low δ^15^N_consist_ | - | - | - | - | - | - | - | 6 | 0.78 | 1 | -0.1 | 2.3 |
|  | High δ^13^C_consist_, High δ^15^N_consist_ | - | - | - | - | - | - | - | 26 | -0.51 | 1 | -2.1 | 0.9 |
| **Note:** Bold *p*-values indicate statistical significance (*p* < 0.05). Asterisks (*) denote significant terms. The model shows poor predictive performance (Testing *R*² = 0.03). δ^15^N_consist_ segment and the δ^13^C_consist_ × δ^15^N_consist_ interaction are the only significant predictors. Segment interaction accounts for the largest relative effect (33.45%). Training *R*² = 0.19 suggests potential overfitting. Dashes (-) indicate statistics not applicable for that parameter type. | | | | | | | | | | | | | |

**Table S9.** Bivariate segmented regression results (PFOS)

| **Component** | **Parameter** | **Value** | **SE** | ***t*** | ***p*** | **Training** | **Testing** | **% Effect** | ***n*** | **Mean** | **SD** | **Min** | **Max** |
| --- | --- | --- | --- | --- | --- | --- | --- | --- | --- | --- | --- | --- | --- |
| **Model Info** | Response Variable | Z_PFOS_ | - | - | - | - | - | - | - | - | - | - | - |
|  | Predictor 1 | δ^13^C_consist_ | - | - | - | - | - | - | - | - | - | - | - |
|  | Predictor 2 | δ^15^N_consist_ | - | - | - | - | - | - | - | - | - | - | - |
|  | Training Observations | 89 | - | - | - | - | - | - | - | - | - | - | - |
|  | Testing Observations | 23 | - | - | - | - | - | - | - | - | - | - | - |
| **Performance** | *R*² | - | - | - | - | 0.35 | -0.05 | - | - | - | - | - | - |
|  | MSE | - | - | - | - | 0.63 | 1.09 | - | - | - | - | - | - |
|  | RMSE | - | - | - | - | 0.79 | 1.04 | - | - | - | - | - | - |
| **Breakpoints** | δ^13^C_consist_ | 0.19 | - | - | - | - | - | - | - | - | - | - | - |
|  | δ^13^C_consist_ Below | 80 (71.4%) | - | - | - | - | - | - | - | - | - | - | - |
|  | δ^13^C_consist_ Above | 32 (28.6%) | - | - | - | - | - | - | - | - | - | - | - |
|  | δ^15^N_consist_ | 0 | - | - | - | - | - | - | - | - | - | - | - |
|  | δ^15^N_consist_ Below | 71 (63.4%) | - | - | - | - | - | - | - | - | - | - | - |
|  | δ^15^N_consist_ Above | 41 (36.6%) | - | - | - | - | - | - | - | - | - | - | - |
| **Coefficients** | Intercept | -0.61 | - | - | - | - | - | - | - | - | - | - | - |
|  | δ^13^C_consist_ | -0.45 | 0.2 | -2.8 | **0.01**** | - | - | 17.91 | - | - | - | - | - |
|  | δ^13^C_consist_ segment | 1.26 | 0.5 | 2.56 | **0.01*** | - | - | 50.55 | - | - | - | - | - |
|  | δ^15^N_consist_ | 0.03 | 0.2 | 0.19 | 0.85 | - | - | 1.1 | - | - | - | - | - |
|  | δ^15^N_consist_ segment | -0.05 | 0.3 | -0.2 | 0.86 | - | - | 1.93 | - | - | - | - | - |
|  | δ^13^C_consist_ × δ^15^N_consist_ interaction | 0.16 | 0.1 | 1.96 | 0.05 | - | - | 6.44 | - | - | - | - | - |
|  | Segment interaction | 0.55 | 0.7 | 0.85 | 0.4 | - | - | 22.06 | - | - | - | - | - |
| **Segment Combinations** | Low δ^13^C_consist_, Low δ^15^N_consist_ | - | - | - | - | - | - | - | 65 | -0.28 | 1 | -2.3 | 2.9 |
|  | Low δ δ^13^C_consist_, High δ^15^N_consist_ | - | - | - | - | - | - | - | 15 | -0.4 | 0.6 | -1.8 | 0.5 |
|  | High δ^13^C_consist_, Low δ^15^N_consist_ | - | - | - | - | - | - | - | 6 | 0.54 | 0.7 | -0.8 | 1.4 |
|  | High δ^13^C_consist_, High δ^15^N_consist_ | - | - | - | - | - | - | - | 26 | 0.8 | 0.9 | -0.4 | 3.4 |
| **Note:** Bold *p*-values indicate statistical significance (*p* < 0.05). Asterisks denote significance level (* *p* < 0.05, ** *p* < 0.01). The model shows better training performance than PFCA (*R*² = 0.35) but still poor testing performance (*R*² = -0.05). δ^13^C_consist_ and its segment term are highly significant. δ^13^C_consist_ segment accounts for the largest relative effect (50.6%). High δ^13^C_consist_ segments show positive means regardless of δ^15^N_consist_ levels. Dashes (-) indicate statistics not applicable for that parameter type. | | | | | | | | | | | | | |

# References

[1] Zenobio, J. E., Salawu, O. A., Han, Z. & Adeleye, A. S. Adsorption of per- and polyfluoroalkyl substances (PFAS) to containers. *Journal of Hazardous Materials Advances* **7**, 100130 (2022). <https://doi.org:https://doi.org/10.1016/j.hazadv.2022.100130>

[2] Hansen, K. J., Clemen, L. A., Ellefson, M. E. & Johnson, H. O. Compound-Specific, Quantitative Characterization of Organic Fluorochemicals in Biological Matrices. *Environ. Sci. Technol.* **35**, 766-770 (2001). <https://doi.org:10.1021/es001489z>

[3] Yeung, L. W. Y. *et al.* Perfluorooctanesulfonate and Related Fluorochemicals in Human Blood Samples from China. *Environ. Sci. Technol.* **40**, 715-720 (2006). <https://doi.org:10.1021/es052067y>

[4] Armbruster, D. A. & Pry, T. Limit of blank, limit of detection and limit of quantitation. *Clin. Biochem. Rev.* **29 Suppl 1**, S49-52 (2008).

[5] Wells, G., Prest, H., & Russ IV, C. W. Signal, Noise, and Detection Limits in Mass Spectrometry. (Santa Clara, CA, USA, 2011).
